# Exportin 4 depletion leads to nuclear accumulation of a subset of circular RNAs

**DOI:** 10.1038/s41467-022-33356-z

**Published:** 2022-10-01

**Authors:** Liang Chen, Yucong Wang, Jiamei Lin, Zhenxing Song, Qinwei Wang, Wenfang Zhao, Yan Wang, Xiaoyu Xiu, Yuqi Deng, Xiuzhi Li, Qiqi Li, Xiaolin Wang, Jingxin Li, Xu Liu, Kunpeng Liu, Jincong Zhou, Kuan Li, Yuchan Liu, Shanhui Liao, Qin Deng, Chao Xu, Qianwen Sun, Shengzhou Wu, Kaiming Zhang, Min-Xin Guan, Tianhua Zhou, Fei Sun, Xiujun Cai, Chuan Huang, Ge Shan

**Affiliations:** 1 Department of Clinical Laboratory, The First Affiliated Hospital of USTC, the CAS Key Laboratory of Innate Immunity and Chronic Disease, School of Basic Medical Sciences, Division of Life Science and Medicine, University of Science and Technology of China, Hefei, 230027 China; 2 School of Life Sciences, Chongqing University, Chongqing, 401331 China; 3 Center of Plant Functional Genomics, Institute of Advanced Interdisciplinary Studies, Chongqing University, Chongqing, 401331 China; 4 School of Optometry and Ophthalmology and the Eye Hospital, Wenzhou Medical University, Wenzhou, Zhejiang 325003 China; 5 State Key Laboratory of Optometry, Ophthalmology, and Visual Science, 270 Xueyuan Road, Wenzhou, Zhejiang 325003 China; 6 Center for Plant Biology, School of Life Sciences, Tsinghua-Peking Center for Life Sciences, Tsinghua University, Beijing, 100084 China; 7 MOE Key Laboratory for Membraneless Organelles and Cellular Dynamics, Division of Life Sciences and Medicine, University of Science and Technology of China, Hefei, 230027 China; 8 Division of Life Science and Medicine, CAS Key Laboratory of Structural Biology, University of Science and Technology of China, Hefei, 230027 China; 9 Analytical and Testing Center, Chongqing University, Chongqing, 400030 China; 10 The Children’s Hospital, Zhejiang University School of Medicine and National Clinical Research Center for Child Health, Zhejiang Provincial Key Lab of Genetic and Developmental Disorder, Institute of Genetics, Zhejiang University School of Medicine, Hangzhou, 310016 China; 11 Department of Cell Biology and Department of Gastroenterology, Sir Run Run Shaw Hospital, School of Medicine, Cancer Center, Institute of Gastroenterology, Zhejiang University, Hangzhou, 310016 China; 12 Sir Run-Run Shaw Hospital, Zhejiang University School of Medicine, Hangzhou, 310016 China; 13 Department of Pulmonary and Critical Care Medicine, Regional Medical Center for National Institute of Respiratory Diseases, Sir Run Run Shaw Hospital, School of Medicine, Zhejiang University, Hangzhou, 310016 China

**Keywords:** Non-coding RNAs, RNA transport, Neurophysiology, Reproductive biology

## Abstract

Numerous RNAs are exported from the nucleus, abnormalities of which lead to cellular complications and diseases. How thousands of circular RNAs (circRNAs) are exported from the nucleus remains elusive. Here, we provide lines of evidence to demonstrate a link between the conserved Exportin 4 (XPO4) and nuclear export of a subset of circRNAs in metazoans. Exonic circRNAs (ecircRNAs) with higher expression levels, larger length, and lower GC content are more sensitive to XPO4 deficiency. Cellular insufficiency of XPO4 leads to nuclear circRNA accumulation, circRNA:DNA (ciR-loop) formation, linear RNA:DNA (liR-loop) buildup, and DNA damage. DDX39 known to modulate circRNA export can resolve ciR-loop, and splicing factors involved in the biogenesis of circRNAs can also affect the levels of ciR-loop. Testis and brain are two organs with high abundance of circRNAs, and insufficient XPO4 levels are detrimental, as *Xpo4* heterozygous mice display male infertility and neural phenotypes. Increased levels of ciR-loop, R-loop, and DNA damage along with decreased cell numbers are observed in testis and hippocampus of *Xpo4* heterozygotes. This study sheds light on the understandings of mechanism of circRNA export and reveals the significance of efficient nuclear export of circRNAs in cellular physiology.

## Abstract (web summary)

This study identifies the evolutionarily conserved Exportin 4 as an essential regulator in the nuclear export of circRNAs. Defective circRNA export results in R-loop formation and DNA damage in cells, as well as testis and neurological defects in mice.

## Introduction

Circular RNAs (circRNAs) are covalently closed single-stranded RNAs that are generated from linear precursors by various mechanisms in eukaryotic cells^1–3^. In animals, there are four subclasses of circRNAs: exonic circRNAs (ecircRNAs), exon-intron circRNAs (EIciRNAs), intron-derived circRNAs, and mitochondria-encoded circRNAs (mecciRNAs)^4–9^. EIciRNAs, which are composed of both exonic and intronic sequences, and ecircRNAs, which are formed by exonic sequences, are generated via backsplicing in the nucleus, and ecircRNAs account for most of the eukaryotic circRNAs^1–5,7^. EcircRNAs predominately localize in the cytoplasm^1–3^. The mechanism via which thousands of ecircRNAs are transported into the cytoplasm remains elusive, despite evidence that suggests that the nuclear export process is regulated by DDX helicases known as *Drosophila* Hel25E and its mammalian homologs DDX39A and DDX39B^10^.

Intracellular transport of proteins and RNAs is mediated by nucleocytoplasmic carriers (receptors)^11–14^. These carriers include importins, which transport cargo from the cytoplasm into the nucleus, and exportins (XPOs), which transport macromolecules from the nucleus into the cytoplasm^11–14^. The XPO family in mammals consists of seven members^11–14^. For RNAs, XPO1 (CRM-1) is involved in the export of some mRNAs in an eIF4E/CRM1-dependent manner^15^, although most mRNAs are exported in a CRM1-independent manner^16^. The DDX39-dependent mRNA export is also thought to be CRM1-independent and Nxf1-dependent^17^. XPO-T, which is also known as XPO3, mediates the export of tRNAs^18,19^. XPO5 exports microRNA precursors^20–22^. However, each exportin is generally not specific for just one category of cargos but often has diverse types of protein and RNA substrates^23,24^. For example, XPO5 also mediates the export of various tRNAs, whereas some microRNA precursors with trimethylguanosine-cap are exported by XPO1^23,24^. Efficient export of nuclear RNAs is essential for cellular homeostasis, and abnormalities in export are associated with a plethora of human diseases, e.g., neurological diseases and cancers^11,25,26^.

In mammals, brain and testis are organs with the highest abundance of ecircRNAs^27–29^. Individual ecircRNAs, such as circSry in mice and circBoule in animals and humans have been shown to play roles in the testis^30^. In mammalian neurons, neuropils are enriched with ecircRNAs, thereby suggesting an active transport and localization mechanism^31^. Knockout of individual circRNAs, for example, the cytoplasmic-enriched and brain-specific circRNA Cdr1as, leads to abnormalities in excitatory synaptic transmission, memory, and exploratory behaviors^32^. There is also a recent argument that based on an evolutionary analysis in mammals, that most circRNAs are likely non-functional and splicing “errors”^33^. On the other hand, dysregulation of circRNAs has also been shown to be associated with an array of human diseases including degenerative diseases such as Alzheimer’s disease^34^, and changes of circRNA levels are associated with physiological processes such as ageing^29,35^.

In this study, we identified the conserved Exportin 4 (XPO4) as an essential regulator modulating nuclear export of a subset of ecircRNAs by screening known exportins in mammalian and fruit fly cells. Further experiments were performed to demonstrate the roles of XPO4 in nuclear export of ecircRNAs in animal cells. An array of assays revealed that knockout of XPO4 in mammalian cells led to nuclear accumulation of ecircRNAs, which triggered pervasive formation of circRNA: genomic DNA hybrids (circRNA:DNA R-loops, ciR-loops) and DNA damage. Knockdown of XPO4 in Drosophila cells led to similar effects. A sufficient level of XPO4 was critical to mammals, especially for testis and brain, the two organs with the highest abundance of ecircRNAs. XPO4 heterozygous mice demonstrated male infertility, and significantly increased levels of ciR-loops, R-loops, and DNA damage were observed in the testes. XPO4 heterozygous mice also exhibited neurological defects.

## Results

### Screening in mammalian and fruit fly cells identified a function of XPO4 in ecircRNA export

**Fig. 1:**
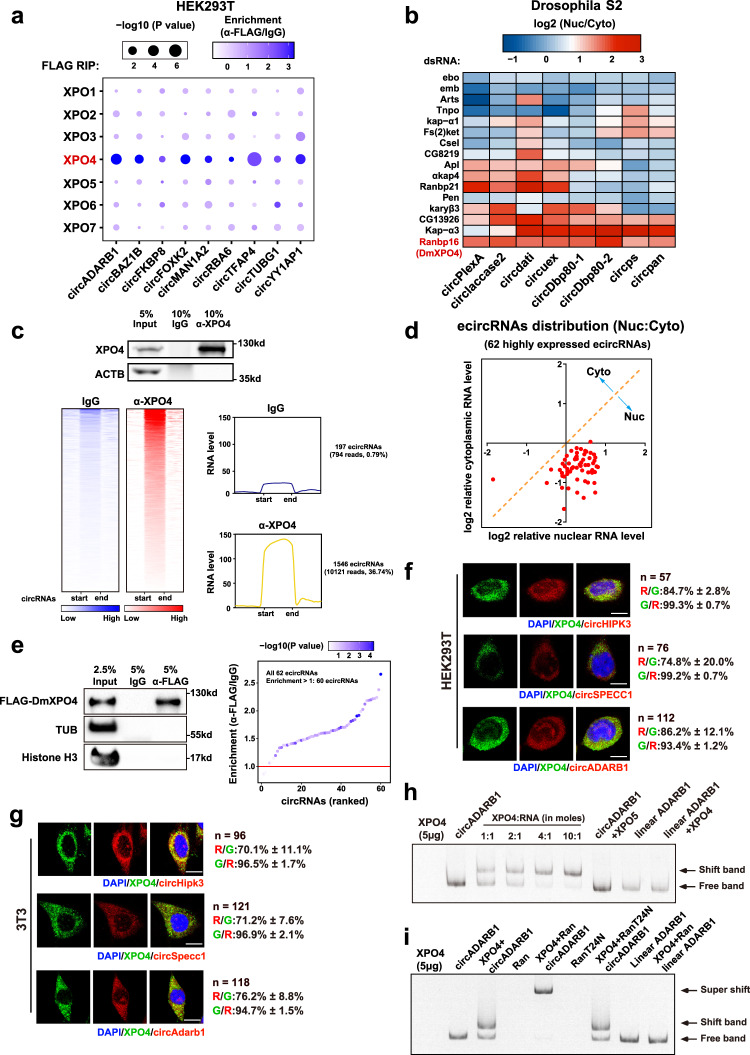
Screening in mammalian and *Drosophila* cells identifies XPO4 in ecircRNA export. **a** Heatmap representation of relative circRNA binding of FLAG-tagged exportins from RNA immunoprecipitation (RIP) in human HEK293T cells. Reverse transcription-quantitative PCR (RT-qPCR) of individual ecircRNA was performed, and the enrichment was normalized to the immunoglobulin G (IgG) control. **b** Heatmap of RNAi screening data using dsRNAs against the 16 factors with nucleocytoplasmic carrier activity in *Drosophila* S2 cells. Nuclear (Nuc) and cytoplasmic (Cyto) levels of individual ecircRNA were analyzed with RT-qPCR, and the Nuc:Cyto ratio of each circRNA was calculated and then normalized to the negative control (Ctrl, no dsRNA empty control). **c** RIP-seq data of ecircRNAs from antibodies (ɑ-XPO4) against the endogenous XPO4 in human HEK293T cells. RIP efficiency of XPO4 protein was validated through Western blot. Heatmaps represent co-precipitated ecircRNAs for XPO4 and IgG control; Metaplots represent RNA level of co-precipitated ecircRNAs for XPO4 and IgG control. Number of ecircRNA reads and proportion of ecircRNA junction reads out of all RNA reads from these loci are shown. RIP-seq data were generated from a combined sample from two biological replicates. **d** Relative changes of Nuc and Cyto levels of 62 highly-expressed ecircRNAs upon dsRNA knockdown of *dmXpo4*. RT-qPCR of individual ecircRNAs was performed, and the corresponding Nuc or Cyto levels of each ecircRNA were normalized to the negative control (Ctrl, no dsRNA empty control). Data are from five independent experiments. **e** DmXpo4-FLAG RIP with antibody against FLAG (α-FLAG). Validation of RIP efficiency is shown with Western blots, and the fold of enrichment for all 62 ecircRNAs was analyzed by RT-qPCR (the enrichment is normalized to IgG control). Data are from three independent experiments. **f**, **g** Fluorescence in situ hybridization (FISH) of circRNAs (red) specified along with immunofluorescence staining (IF) of XPO4 protein (green) in human HEK293T cells and mouse NIH/3T3 cells. The colocalizations between green (XPO4) and red (circRNA) fluorescent signals are shown beside the images. *N* = number of cells analyzed. Scale bars, 20 μm. **h** Electrophoretic mobility shift assay with purified XPO4 protein and in vitro transcribed circADARB1 with indicated molar ratio. XPO5 and linear ADARB1 were used as controls. Arrowheads indicate the shift band and the free band, respectively. **i** Electrophoretic mobility shift assay with purified XPO4 protein and in vitro transcribed circADARB1 in addition with RanGTPase (Ran) and mutant Ran. In **h** and **i**, linear ADARB1 has the same sequences with circADARB1; arrowheads indicate the super-shift band, shift band, and the free band, respectively. Data are representative of two independent experiments. Details of biostatistics and *P* value calculation for **a**, **b**, and **e** are included in the “Quantification and statistical analysis” section of Methods. Source data are provided as a Source data file.

Seeking to identify which XPO protein(s) function in the nuclear export of ecircRNAs, we examined the ecircRNA binding capacities of all seven XPOs in experiments using human HEK293T cells in which FLAG-tagged exportins were assessed using RNA immunoprecipitation (RIP). Briefly, XPO4 exhibited the highest binding capability towards all nine randomly chosen human ecircRNAs in these assays (Fig. 1a). To further evaluate the XPOs that are involved in ecircRNA export in animals, we also conducted RNAi screening with *Drosophila* S2 cells, in which double-stranded RNAs (dsRNAs) were used to individually knock down (KD) all the genes that correspond to the flybase GO term “nucleocytoplasmic carrier activity”, which includes 16 nucleocytoplasmic carrier orthologs (Fig. 1b). KD of Ranbp16, which is the mammalian XPO4 ortholog (henceforth termed “dmXPO4”) (Supplementary Fig. 1a), resulted in obviously elevated nucleus/cytoplasm (Nuc/Cyto) ratio of all eight *Drosophila* ecircRNAs that were examined (Fig. 1b; Supplementary Fig. 1b). We also used antibodies against each mammalian exportin expressed at the endogenous level to perform RIP with HEK293T cells under normal physiological conditions, and the binding capacity of endogenous XPO4 and control exportins towards circRNAs have been examined and compared (Supplementary Fig. 1c). The results from the endogenous proteins were in consistent with the data from Flag-tagged exportins. In the RIP assay with antibody against the endogenous XPO4 protein, linear RNAs with relatively high expression levels such as 18S rRNA, U6 snRNA, U7 snRNA, ACTB mRNA, GAPDH mRNA, and the lncRNA MALAT1 could not be pulled down together with XPO4 protein (Supplementary Fig. 1d). Although we could not exclude the involvement in ecircRNA export of the other factors in these focused screenings without further investigation, our data indicated that XPO4 might play an evolutionarily conserved role in ecircRNA export, and we investigated this protein further.

Antibodies against endogenous XPO4 were then used to perform RIP with human HEK293T cells, and RNA-sequencing (RNA-seq) of the RIP materials revealed that more than 1500 ecircRNAs were pulled down together with XPO4 (Fig. 1c; Supplementary Fig. 1e). In this case, the ~1500 ecircRNAs were underestimated due to limitations in the identification of ecircRNAs by circRNA bioinformatics and RNA-seq with small amount of RIP materials^36^. Further supporting a functional role for dmXPO4 in ecircRNA export, we found that 61 of the 62 highly abundant ecircRNAs^37,38^, showed a statically significantly increased Nuc/Cyto ratio upon dmXPO4 KD in S2 cells (Fig. 1d). FLAG-tagged dmXPO4 also pulled down 60 of the 62 ecircRNAs examined in the RIP experiments (Fig. 1e). Fluorescent in situ hybridization (FISH) of ecircRNAs (using three examples: circHipk3, circSpecc1, and circAdarb1) and immunofluorescence (IF) staining against the XPO4 protein in HEK293T and 3T3 cells revealed that more than 93% XPO4 signals overlapped with ecircRNAs (Fig. 1f and g; Supplementary Fig. 1f, g), thereby suggesting that the XPO4 protein might have some chance to interact with ecircRNAs in mammalian cells. Then, the binding of XPO4 to ecircRNAs was examined by an electrophoretic mobility shift assay (EMSA) with circADARB1 as an example of an ecircRNA (Fig. 1h). XPO4 shifted with circADARB1 but not the linear RNA of the same sequence; XPO5, which was used as a negative control of XPO4, did not bind to circADARB1 (Fig. 1h). Similar to the other exportins, XPO4 works together with RanGTPase^39^, and in a supershift assay, XPO4 and RanGTPase complex demonstrated efficient binding to the circRNA tested (Fig. 1i). These results identified XPO4 as a conserved factor in animals that functions in the nuclear export of ecircRNAs.

### Knockout of XPO4 resulted in nuclear accumulation of ecircRNAs

**Fig. 2:**
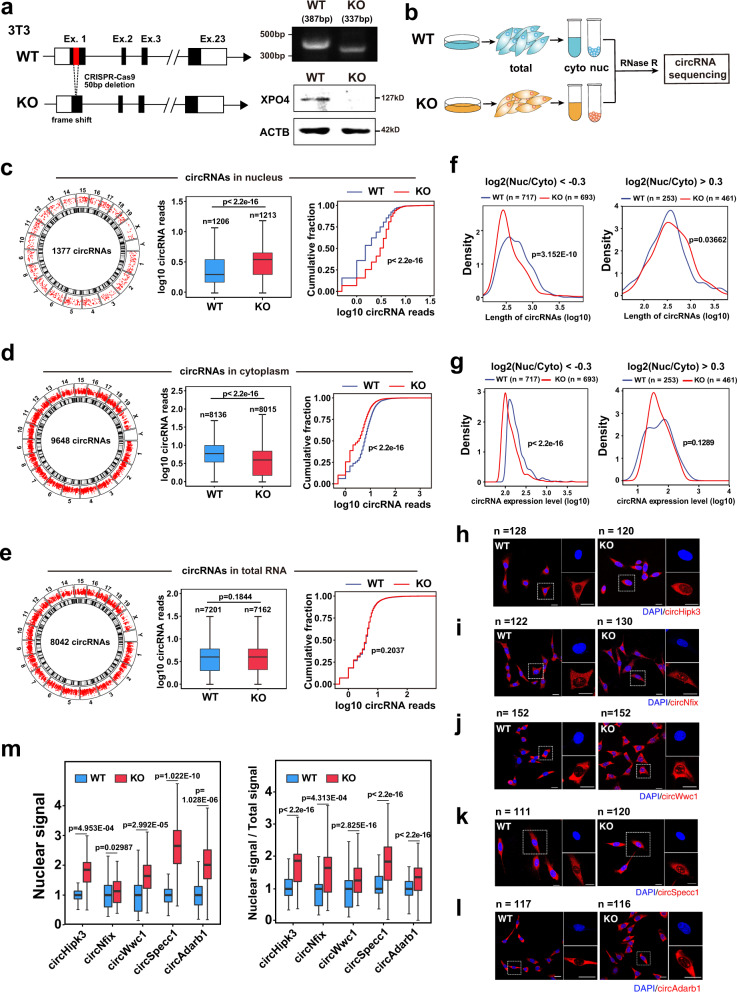
XPO4 knockout results in nuclear accumulation of ecircRNAs. **a** Scheme for generating the NIH/3T3 XPO4 CRISPR-cas9 knockout (KO) cells. Validation with genomic PCR and western blot of the successful KO in NIH/3T3 cells are also shown. Data are representative of three independent experiments. **b** Workflow of nucleocytoplasmic separation and sample preparation of circRNA sequencing. **c**, **d**, **e** Bioinformatics analyses of ecircRNAs in nuclear, cytoplasmic, and total RNAs from the wild-type (WT) and KO cells. Number and genomic distribution of detected ecircRNAs are shown in circus plot. The corresponding ecircRNA reads from WT and XPO4 KO cells are compared in the middle. Cumulative fraction curves demonstrating the ecircRNA levels in the XPO4 KO and WT cells are shown to the right. *n* = number of circRNAs analyzed. **f** circRNA length distributions of WT and XPO4 KO cells relatively enriched in the cytoplasmic fraction (log2(Nuc/Cyto) < −0.3), and nuclear fraction (log2(Nuc/Cyto) > 0.3), respectively. **g** circRNA expression levels of WT and XPO4 KO cells in the cytoplasmic fraction (log2(Nuc/Cyto) < −0.3), and nuclear fraction (log2(Nuc/Cyto) > 0.3), respectively. **h**, **i**, **j**, **k**, **l** Representative FISH images of ec**i**rcRNA (Red) in NIH/3T3 WT and XPO4 KO cells. *n* = number of cells analyzed. Scale bars, 20 μm. **m**, Quantification of nuclear FISH signals and the ratio of nuclear: total signals of the ecircRNA analyzed in **h**, **i**, **j**, **k** and **l**. For **c**–**e**, **m**, boxes for box-plot graphs extend from the 25th to 75th percentiles and the line in the middle is plotted at the mean. Whiskers delineate all data points from minimum to maximum. For **c**–**g**, **m**, *P* values of boxplots are from unpaired two-sided Student’s *t* test. For **c**–**e**, *P* values of cumulative frac*t*ion curves are from the Kolmogorov–Smirnov test. Data are shown as means ± SD. *P* values were indicated in the figures. Source data are provided as a Source data file.

We constructed a mouse XPO4 knockout (KO) cell line in 3T3 embryonic fibroblasts (Fig. 2a), after which the total, nuclear, and cytoplasmic RNAs from the KO and wild-type (WT) cells were isolated, treated with RNase R (to digest linear RNAs)^40^, and subjected to RNA-seq (Fig. 2b and Supplementary Fig. 1h). By bioinformatics analysis of RNA-seq data, we found that compared to the WT cells, the KO cells had significantly increased ecircRNA levels in the nuclear fraction (Fig. 2c) and significantly decreased ecircRNA levels in the cytoplasm (Fig. 2d). Notably, there was no significant difference in total ecircRNA level between the WT and XPO4 KO cells (Fig. 2e). The nuclear levels of EIciRNAs, which are a subclass of circRNAs that are also generated from backsplicing in the nucleus and have been previously shown not to be exported into the cytoplasm^4^, were not affected by XPO4 KO (Supplementary Fig. 1i). These results demonstrated that XPO4 KO led to nuclear accumulation of ecircRNAs and did not affect the overall backsplicing of circRNA biogenesis.

We then examined the expression levels, length distribution, GC content, and ΔG distributions of the ecircRNAs significantly enriched in the cytoplasm (log2(Nuc/Cyto) <−0.3, *P* < 0.05; defined here as cytoplasmic ecircRNAs) or in the nuclear (log2(Nuc/Cyto) > 0.3, *P* < 0.05; defined here as nuclear ecircRNAs) in the WT and XPO4 KO cells (Fig. 2f, g and Supplementary Fig. 1j, k). The cytoplasmic ecircRNAs in WT cells were relatively XPO4 sensitive ecircRNAs (717 ecircRNAs, ~37% of all cytoplasmic ecircRNAs), and the cytoplasmic ecircRNAs in XPO4 KO cells were relatively insensitive to XPO4 deletion (693 ecircRNAs, ~36% of all cytoplasmic ecircRNAs). Cytoplasmic ecircRNAs in the WT cell demonstrated significantly larger length and higher expression levels than those in the XPO4 KO cells (Fig. 2f, g). Nuclear ecircRNAs in the WT cell demonstrated significantly smaller length, although no statistical difference in expression levels, compared to those in the XPO4 KO cells (Fig. 2f, g). The G/C content was lower, and in correlation with this, the △G was higher for cytoplasmic ecircRNAs in the WT cells (Supplementary Fig. 1j). The nuclear ecircRNAs of the WT cells and the KO cells did not show significant difference in the GC content or △G (Supplementary Fig. 1k). These results together indicated that ecircRNAs with larger length, higher expression levels, lower G/C content (and thus may be less complex structure) were more sensitive to the deficiency of or more depended on XPO4.

We also suspected the existence of an XPO4-independent mechanism of ecircRNA nuclear/cytoplasmic distribution because even in the XPO4 KO cells, most ecircRNAs were still cytoplasmic. One possible mechanism was a passive nucleus-to-cytoplasm process in which ecircRNAs in dividing cells might diffuse out of the nucleus during the breakage of the nuclear envelope. In support this possibility, all three ecircRNAs that we examined showed higher nuclear levels in cells before nuclear envelope breakdown (G2/M stage) than in cells with mix stages (Supplementary Fig. 1l). To observe the nuclear accumulation of individual ecircRNAs, we performed FISH assays, and five ecircRNAs, namely, circHipk3, circSpecc1, circNfix, circWwc1, and circAdarb1 were randomly chosen for imaging. Each of these ecircRNAs had significantly increased nuclear signals and Nuc/Cyto ratio in the XPO4 KO cells compared to the WT cells (Fig. 2h–m).

Previous studies in mammalian cells showed that XPO4 functions in the nuclear export of eIF5A, a translation elongation factor^41^. Perturbation of eIF5A would lead to translational defects that could have unforeseen effects on ecircRNA export. To examine the possibility that the functional involvement of XPO4 in ecircRNA export might be related to eIF5A, we either overexpressed or knocked down eIF5A in WT cells and XPO4 KO cells (Supplementary Fig. 2a–d). There was no significant change in the Nuc/Cyto ratio for any of the four ecircRNAs that were examined under eIF5A overexpression or knockdown in either WT or XPO4 KO cells (Supplementary Fig. 2a–d). We also examined Smad3, another known cargo of XPO4^42^. There was also no significant change in the Nuc/Cyto ratio for any of the four ecircRNAs that were examined under Smad3 overexpression or knockdown in either WT or XPO4 KO cells (Supplementary Fig. 2e–h). Another cargo of XPO4, SOX2 is a key transcription factor required for pluripotency, and is not expressed in differentiated cells such as 3T3^43^, and thus SOX2 might not be relevant to the cellular phenotypes upon XPO4 KD or KO. Disturbing the mRNA export and translation in cells through eIF4E knockdown or overexpression also had no association with circRNA cyto/nuclear localization (Supplementary Fig. 2i–l). In addition, there was no significant change in the nuclear/cytoplasmic distribution of several RNAs with high abundance such as 18S rRNA, GAPDH mRNA, ACTB mRNA, U6 snRNA, U7 snRNA, and the lncRNA Malat1 upon the depletion of XPO4 (Supplementary Fig. 1h), and XPO4 did not bind to these RNAs (Supplementary Fig. 1d). However, we cannot rule out that besides impaired nuclear export of ecircRNAs, the depletion of XPO4 disrupts the export of other types of RNAs directly or indirectly, as XPO4 is a multi-functional exportin.

Taken together, we demonstrated a subset of ecircRNAs with relatively higher expression levels, larger length, and lower GC content were sensitive to XPO4 depletion. Deficiency of XPO4 led to nuclear accumulation of ecircRNAs.

### Nuclear-accumulated ecircRNAs generated R-loops and DNA damage

**Fig. 3:**
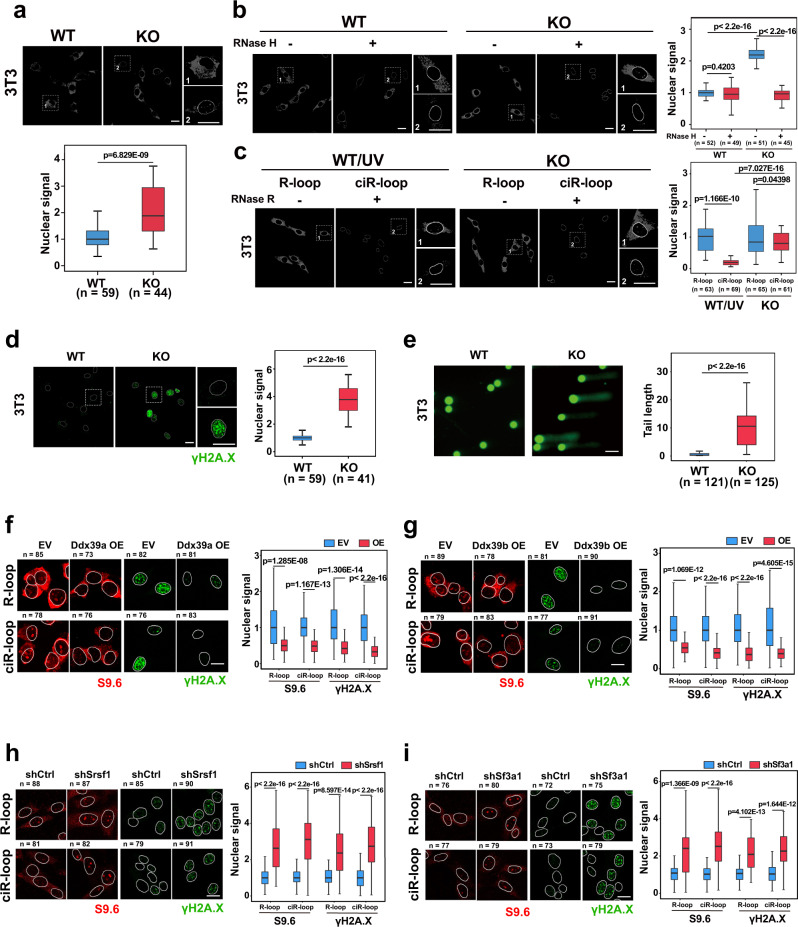
Nuclear-accumulated ecircRNAs generate ciR-loops, R-loops, and DNA damage. **a** Immunofluorescent staining (IF) of R-loop with the antibody S9.6 in NIH/3T3 WT and XPO4 KO cells in gray-scale images. Representative images and enlarged regions are shown. Quantification of nuclear IF signals is shown with bar figure. **b** IF of R-loop with or without the RNase H digestion in NIH/3T3 WT and XPO4 KO cells. Representative gray-scale images and enlarged regions are shown. Quantification of nuclear IF signals is shown with bar figure. **c** IF of R-loop, ciR-loop in NIH/3T3 WT and XPO4 KO cells. Representative in gray-scale images and enlarged regions are shown. WT treated with UV (WT/UV) was used for comparison. Quantification of nuclear IF signals is shown with bar figure. **d** IF of DNA damage with the antibody against γH2A.X in NIH/3T3 WT and XPO4 KO cells. Representative images and enlarged regions are shown (DAPI, blue; R-loop, red; DNA damage, green). Quantification of nuclear IF signals is shown with bar figure. **e** Comet assay in NIH/3T3 WT and XPO4 KO cells. Representative comet assay images of γH2A.X (DNA damage) are shown (DAPI, blue; DNA damage, green). Quantification of relative tail length is shown. **f** IF of R-loop, ciR-loop, and DNA damage upon Ddx39a overexpression (OE) in XPO4 KO cells. Corresponding empty vector (EV) was used as a negative control. R-loop, red; DNA damage, green. Quantification of nuclear IF signals is shown with bar figure. **g** IF of R-loop, ciR-loop, and DNA damage upon Ddx39b overexpression (OE) in XPO4 KO cells. A corresponding empty vector (EV) was used as a negative control. R-loop, red; DNA damage, green. Quantification of nuclear IF signals is shown with bar figure. **h** IF of R-loop, ciR-loop, and DNA damage upon Srsf1 shRNA knockdown (KD) in NIH/3T3 WT cells. Corresponding empty vector (shCtrl) was used as a negative control. R-loop, red; DNA damage, green. Quantification of nuclear IF signals is shown with bar figure. **i** IF of R-loop, ciR-loop, and DNA damage upon Sf3a1 KD in NIH/3T3 WT cells. Corresponding empty vector (shCtrl) was used as a negative control. R-loop, red; DNA damage, green. Quantification of nuclear IF signals is shown with bar figure. For **a**–**i**, *n* = number of cells analyzed. Scale bars, 10 μm. Boxes for box-plot graphs extend from the 25th to 75th percentiles and the line in the middle is plotted at the mean. Whiskers delineate all data points from minimum to maximum. Data are shown as means ± SD. *P* values from unpaired two-sided Student’s *t* test, *P* values were indicated in the figures. Source data are provided as a Source data file.

Next, we investigated potential downstream impacts of the nuclear accumulation of ecircRNAs in XPO4 KO cells. Defects in RNA export factors often lead to unscheduled or deleterious formation of RNA:DNA hybrids (R-loops)^44^. R-loops can be detected and studied with the broadly used monoclonal antibody S9.6^45^. IF staining with S9.6 antibody showed a significantly increased nuclear signal intensity in the KO vs. WT cells (Fig. 3a). Note that the S9.6 antibody is known to give unspecific cytoplasmic IF signals^46,47^, we therefore focused on the nuclear signals and tested whether the nuclear signals were from R-loops. We found that pretreatment of RNase H, which specifically digests the RNA in the R-loops^48^, largely eliminated the nuclear S9.6 IF signals, thereby confirming that these nuclear signals were from R-loops (Fig. 3b). In addition, RNase H treatment also removed most of the cytoplasmic IF signal (Fig. 3b). The IF signal in the cytoplasm might potentially be attributed to mitochondrial R-loops^49^, which would be removed upon RNase H treatment as well. The R-loop levels in KO cells were even comparable to those in WT cells under UV exposure, which is a treatment that is known to generate DNA damage and the associated R-loops (Fig. 3c)^50^. Pretreatment with RNase R, which is an exonuclease that specifically digests linear RNAs but not circRNAs^40,51^, eliminated the nuclear S9.6 IF signals in UV-exposed WT cells (Fig. 3c). This result indicated that the R-loops that were generated under UV treatment were formed by linear RNAs (liR-loops), which also served as a control for determining the effectiveness of RNase R digestion. A significant proportion of R-loop signals remained for XPO4 KO cells after the RNase R treatment (Fig. 3c), and this result indicated that the majority of R-loops in the XPO4 KO cells were formed by circRNAs, which we termed circRNA R-loops (ciR-loops).

The presence of extensive R-loops is known to trigger DNA damage;^52^ consistently, our IF analysis showed that KO cells had significantly stronger nuclear signals for the DNA damage marker γH2A.X (Ser-139 phosphorylated histone H2A.X) than WT cells (Fig. 3d). The comet assay, which is used to evaluate DNA damage in individual cells^53^, also revealed a substantial increase in DNA damage in the XPO4 KO cells (Fig. 3e). In addition, eIF5A, Smad3, or eIF4E had no role in the formation of R-loops or DNA damage in this context, as neither knockdown of eIF5A, Smad3, or EIF4E in the WT cells nor overexpression of eIF5A, Smad3, or eIF4E in the KO cells had a significant effect on R-loop formation or DNA damage (Supplementary Fig. 3a–f). In fly S2 cells, knockdown of dmXPO4 also resulted in increased nuclear R-loops and ciR-loops (Supplementary Fig. 3g, h). The comet assay and IF against γH2A.V (the *Drosophila* γH2A.X ortholog) demonstrated that knockdown of dmXPO4 led to significantly higher levels of DNA damage (Supplementary Fig. 3i, j).

DDX39A and DDX39B have been shown to regulate the nuclear export of ecircRNAs^10^. A recent study demonstrated that DDX39B could unwind R-loops^54^. We assessed the possibility that DDX39 participated in ecircRNA export by resolving ciR-loops. We overexpressed Ddx39a and Ddx39b separately in XPO4 KO cells, or their ortholog Hel25E in Drosophila DmXPO4 knockdown cells and found that Ddx39a, Ddx39b, or Hel25E overexpression led to decreased R-loop and ciR-loop levels, as well as decreased DNA damage levels (Fig. 3f, g; Supplementary Fig. 4a). It has been reported that more ecircRNAs can be generated from backsplicing when core components of splicing machineries, such as U2 snRNP and SR proteins, are limited^55^. Then, we individually knocked down the expression of SF3A1 (a U2 snRNP factor) or SRSF1 (an SR protein), to cause “overexpression” of ecircRNAs by promoting their biogenesis. The results showed that knockdown of either SF3A1 or SRSF1 led to increased levels of the four ecircRNAs examined, and concurrently significant escalations of R-loops, ciR-loops, and DNA damage in 3T3 cells (Fig. 3h, i and Supplementary Fig. 4b). Knockdown of *Drosophila* SF3A1 also led to increased levels of the eight ecircRNAs examined and augmented levels of R-loops, ciR-loops, and DNA damage in S2 cells (Supplementary Fig. 4c, d).

Detrimental R-loops can trigger DNA double-strand breaks (DSBs), while R-loops can be actively induced at DSBs^52,56^. We already showed that DNA damage that was caused by UV exposure was associated with liR-loops but not the ciR-loops (Fig. 3c). We also generated DNA damage via an alternative approach by knocking down the expression of replication protein A (RPA), which is the major protein that binds and protects single-stranded DNA (ssDNA) in eukaryotic cells (Supplementary Fig. 4e, f). Knockdown of RPA caused the known effect of DNA damage and concurrently increased the level of liR-loops but not the level of ciR-loops (Supplementary Fig. 4g). Furthermore, the two ecircRNAs, namely, circHipk3 and circSpecc1, which we surveyed did not show nuclear accumulation upon RPA knockdown (Supplementary Fig. 4h). These results indicated that DNA damage induced liR-loop but not ciR-loop formation and that the ciR-loops in XPO4 KO cells were formed by the nuclear-accumulated ecircRNAs.

We also examined the *C. elegans* XPO4 ortholog *Y69A2AR.16* (*ceXPO4*) (Supplementary Figs. 1a and 5a), and *ceXPO4* KO worms demonstrated decreased brood size and slower development (Supplementary Fig. 5b–d). At the molecular and cellular levels, the KO worms had slightly but statistically significant increased levels of circRNAs, stronger signals for R-loops and significantly increased DNA damage in the germline compared to wild type N2 worms (Supplementary Fig. 5e–g).

Collectively, these results indicated that insufficient ecircRNA export due to the lack of XPO4 might lead to evolutionarily conserved cellular phenotypes of R-loop accumulation and DNA damage. We deduced that the shortage of XPO4 protein led to nuclear accumulation of ecircRNAs, which then formed deleterious ciR-loops. Although perturbation of major regulatory factors such as XPO4, splicing factors, or DDX39A/B might cause complex effects, and the cellular phenotype of increased R-loops upon the knockdown or knockout of these factors provided only correlative evidence that the ecircRNAs contribute in a significant way with R-loop and ciR-loop formation. As for the links between R-loop & ciR-loop formation and DNA damage, the characterizations below might provide further clues.

### Pervasive formation of *trans* ciR-loops was positively correlated with liR-loops in XPO4 KO cells

**Fig. 4:**
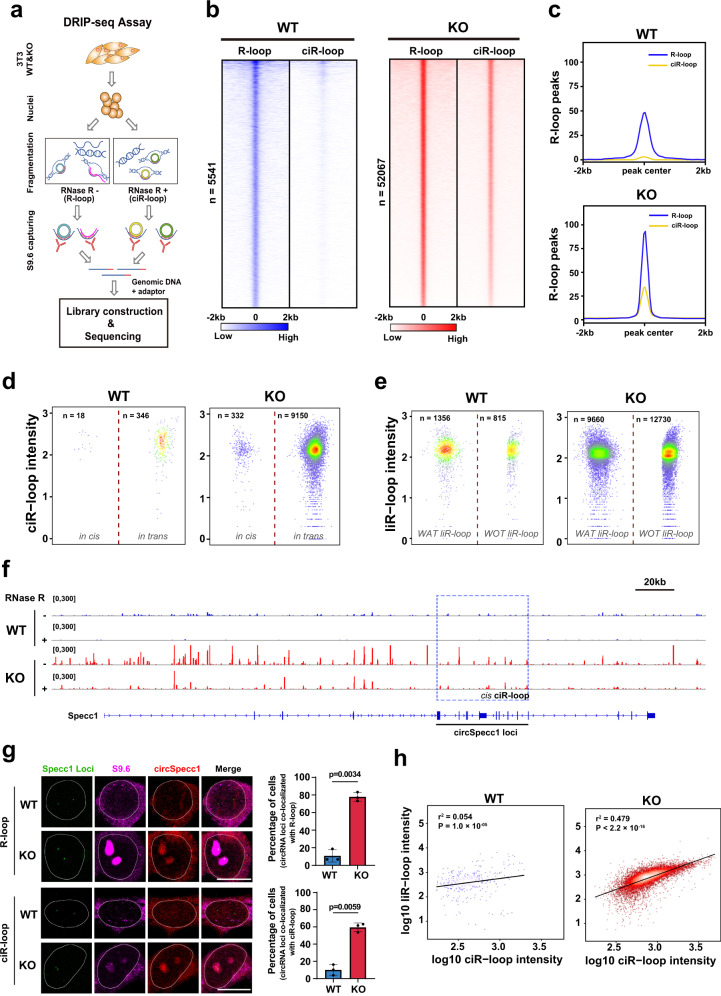
Pervasive formation of *trans* ciR-loops that positively correlates with liR-loops in XPO4 KO cells. **a** Schematic illustration of DNA–RNA immunoprecipitation (DRIP) for R-loops and ciR-loops. **b** Heatmap demonstration of the density of R-loop DRIP-seq reads in WT and KO cell with or without RNase R digestion. **c** Read-counts density pileups of R-loop and ciR-loop peaks in WT and KO cell. **d** Scatterplot demonstration of the ciR-loop distribution (Definitions of in trans and in cis ciR-loop are provided in “Methods”). Each dot represents one ciR-loop detected. Color intensity indicates local point density. Numbers of ciR-loop are indicated. **e** Scatterplot demonstration of the liR-loop distribution. Each dot represents one liR-loop identified. Color intensity indicates local point density. The number of liR-loop is indicated. Definitions of WAT liR-loop and WOT liR-loop are provided in “Methods”. **f** Snapshot of Specc1 genomic region with R-loops and ciR-loops detected in WT and KO cells. Blue dashed box shows the genomic region that encodes circSpecc1. **g** FISH assay for Specc1 genomic loci, circSpecc1, and IF assay for R-loop in WT and KO cells. The bar figure shows percentage of cells with Specc1 genomic loci co-localizing with R-loop or ciR-loop signals. Scale bars, 10 μm. Dots represent three independent experiments. Data are shown as mean ± SD. *P* values from unpaired two-sided Student’s *t* test. **h** Correlation of ciR-loop and liR-loop intensity in WT and KO cells at overlapping genomic regions, respectively. R square and *P* value are from two-sided correlation test, and reference lines are indicated. Details of correlation analysis are provided in “Methods”. Source data are provided as a Source data file.

To investigate the genome-wide features of R-loops, we performed DNA:RNA hybrid immunoprecipitation sequencing (DRIP-seq)^57^, in which the DNA strands of the R-loops were sequenced. The total R-loop profiles were acquired from DRIP-seq using samples without RNase R treatment, and the ciR-loop profiles were obtained from DRIP-seq using samples with RNase R predigestion (Fig. 4a). The liR-loop profiles were deduced by subtracting the ciR-loops from the total R-loop profiles.

In the WT cells, low levels of R-loops, which consisted primarily of liR-loops, were discovered (Fig. 4b, c). The XPO4 KO cells contained ~9.5 times more R-loops than the WT cells, and both ciR-loops and liR-loops contributed to the pervasive R-loop formation (Fig. 4b, c). The liR-loops were positively associated with genes with relatively higher expression levels, especially in the WT cells (Supplementary Fig. 6a, b). This phenomenon was consistent with the normal physiological formation of R-loops during the process of transcription^58–62^. In both the WT and KO cells, ciR-loops showed no correlation with the gene expression levels (Supplementary Fig. 6c, d). Next, we analyzed ciR-loops based on their genomic positions. ciR-loops that were detected in genomic region that did not generate circRNAs were considered as ciR-loops that were formed in trans; otherwise, they were regarded as *cis* ciR-loops (see “Methods” for details). In the KO cells, increases in ciR-loops were due to the accumulation of both *cis* and *trans* ciR-loops, and the ciR-loops that were formed in trans in the KO cells were predominant with high absolute (over 9000) and relative (an increase over 29 times) levels (Fig. 4d). We divided the liR-loops into two subtypes: those that formed at genomic loci with and without known annotated mouse transcripts (see “Methods” for details). Both liR-loops from genomic loci with annotated transcripts (WAT liR-loops) and those from genomic loci without annotated transcripts (WOT liR-loops) were greatly increased in the KO cells, and the increase of WOT liR-loops in the KO cells was pervasive, with enormous absolute (over 12,000) and relative (an increase over 15 times) levels (Fig. 4e). For example, R-loops and ciR-loops that formed along the genomic region of Specc1 were demonstrated (Fig. 4f). ciR-loops formed in cis by circSpecc1 and presumably in trans by circRNAs that were not encoded by Specc1 demonstrated substantial augmented signals in the KO cells (Fig. 4f). Here the *cis* or *trans* was determined computationally, and some cautions might be necessary for the interpretation (Fig. 4f). Similar to the global features, liR-loops also showed a significant increase in the Specc1 genomic region (Fig. 4f). Examination of FISH signals of Specc1 genomic DNA and circSpecc1 RNA and of IF staining of R-loops and ciR-loops demonstrated consistent results (Fig. 4g). Furthermore, in KO but not in WT cells, ciR-loops and liR-loops were positively correlated at overlapping genomic loci (Fig. 4h).

Based on these global features, the nuclear-accumulated ecircRNAs in the XPO4 KO cells produced predominately *trans* ciR-loops and concurrent pervasive formation of liR-loops. A possibility required some concern here was that the levels of ciR-loops were so high in the XPO4 KO cells, that they might relatively be overrepresented in the high-throughput DRIP sequencing than ciR-loops in the WT cells, especially at the RNase R-minus condition. Along with previous imaging results of R-loops and DNA damage, these data suggested that nuclear accumulation of ecircRNAs under insufficient XPO4 caused the formation of ciR-loops that eventually generated DNA damages and that DNA breakage then presumably drove the formation of WOT liR-loops. However, we could not rule out that besides impaired nuclear export of ecircRNAs, the depletion of XPO4 might cause other unknown disrupts that also contributed to the DNA damage.

### *Xpo4*^*+/−*^ mice exhibit testis defects

**Fig. 5:**
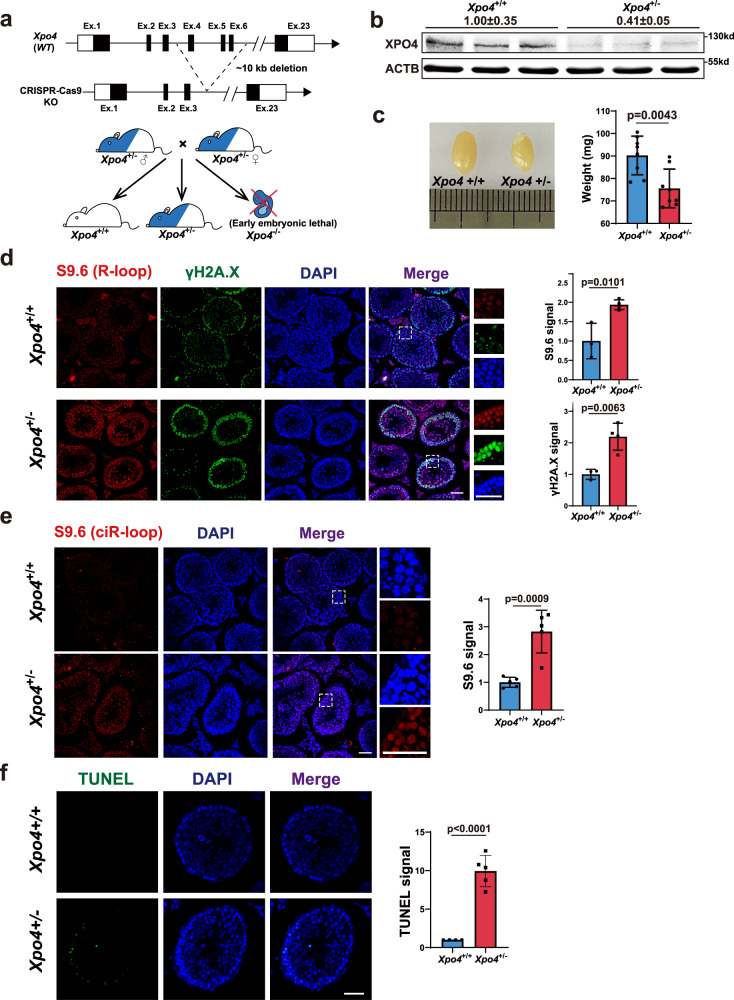
Insufficient XPO4 dosage causes testis deficits in *Xpo4*^*+/−*^ mice. **a** Scheme for generation of CRISPR-cas9 *Xpo4* knockout (KO) mouse. *Xpo4*^*−/−*^ homozygotes were early embryonic lethal. **b** XPO4 protein levels of *Xpo4*^*+/+*^ and *Xpo4*^*+/−*^ mice. **c** Representative images of testes of *Xpo4*^*+/+*^ and *Xpo4*^*+/−*^ mice. Quantification of testis weight is shown. *n* = 8 (*Xpo4*^*+/+*^) and 8 (*Xpo4*^*+/−*^). **d** Representative IF images with enlarged regions of R-loop and γH2A.X (DNA damage) in testes of *Xpo4*^*+/+*^ and *Xpo4*^*+/−*^ mice (DAPI, blue; R-loop, red; DNA damage, green). Representative images and quantification are shown. *n* = 3 (*Xpo4*^*+/+*^) and 4 (*Xpo4*^*+/−*^). **e** Representative IF images with enlarged regions of ciR-loop in testes of *Xpo4*^*+/+*^ and *Xpo4*^*+/−*^ mice. Representative images and quantification are shown. *n* = 5 (*Xpo4*^*+/+*^) and 5 (*Xpo4*^*+/−*^). **f** TUNEL assay in testes of *Xpo4*^*+/+*^ and *Xpo4*^*+/−*^ mice. Representative images and quantification are shown. DAPI, blue; TUNEL, green. *n* = 4 (*Xpo4*^*+/+*^) and 5 (*Xpo4*^*+/−*^). Scale bars, 50 μm (**d**–**f**). For **c**–**f**, data are shown as means ± SD. *P* values from unpaired two-sided Student’s *t* test, *P* values were indicated in the figures. Source data are provided as a Source data file.

Next, we assessed the physiological relevance of ecircRNA export using *Xpo4* CRISPR knockout mice (Fig. 5a). *Xpo4*^*−/−*^ homozygotes were early embryonically lethal, at least before D10.5; thus, we focused on *Xpo4*^*+/−*^ heterozygotes in the following investigations (Fig. 5a, b).

An evident phenotype of *Xpo4*^*+/−*^ was that about half of the *Xpo4*^*+/−*^ males were infertile (Supplementary Fig. 7a). The testes of *Xpo4*^*+/−*^ mice were significantly smaller than those of *Xpo4*^*+/+*^ mice (Fig. 5c). Sperm of *Xpo4*^*+/−*^ mice displayed abnormalities such as coiled/winding flagella, irregular shapes, and missing head (Supplementary Fig. 7b). When examined with computer-assisted sperm analysis (CASA), sperm of *Xpo4*^*+/−*^ mice, compared to *Xpo4*^*+/+*^ sperm, had much less progressive cells and motile cells (Supplementary Fig. 7c). Hematoxylin and eosin (H&E) staining of testis slices also revealed that seminiferous tubules in *Xpo4*^*+/−*^ mice were significantly smaller (Supplementary Fig. 7d). H&E staining of epididymis showed that about half of the *Xpo4*^*+/−*^ epididymal lumen were with dramatically decreased amount of sperm or almost empty (Supplementary Fig. 7e). R-loop and DNA damage levels were much higher, and the levels of ciR-loop were significantly increased in the testes of *Xpo4*^*+/−*^ mice (Fig. 5d, e). There was a dramatic increase in cell death in the testes of *Xpo4*^*+/−*^ mice, as revealed by TUNEL staining (Fig. 5f); it is known that the high level of DNA damage that is triggered by R-loops causes cell death^46,63^. It seems that the testis, which is an organ with a relatively high abundance of circRNAs^29,64^, is very sensitive to low XPO4 levels and the consequent ecircRNA export deficiency.

### *Xpo4*^*+/−*^ mice exhibit neurological defects

**Fig. 6:**
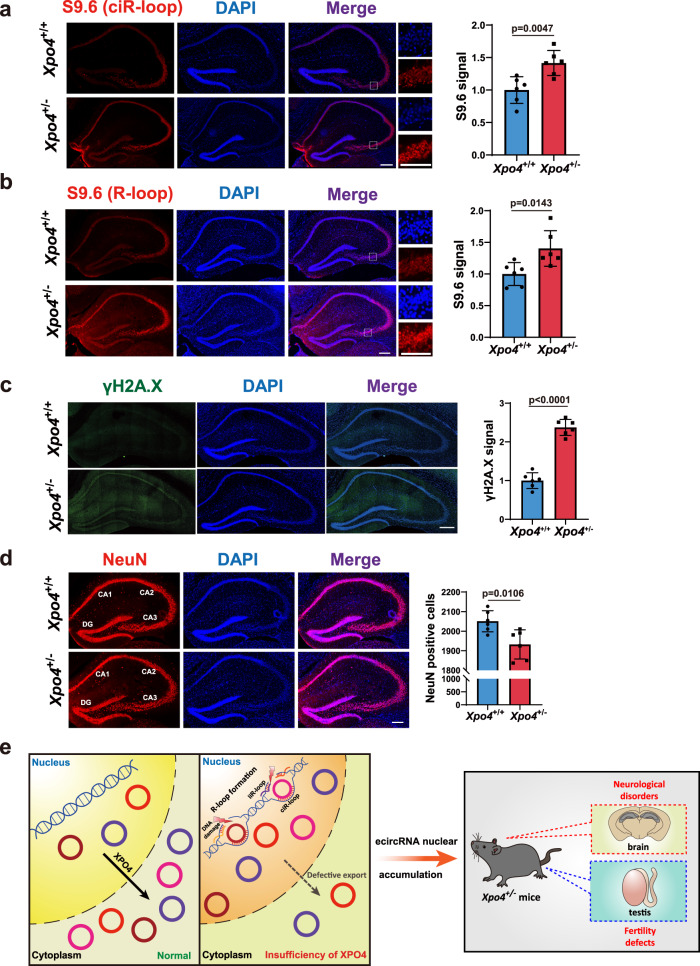
Insufficient XPO4 dosage causes neurological deficits in *Xpo4*^*+/−*^ mice. **a** Representative IF images with enlarged regions of ciR-loop in the hippocampus of *Xpo4*^*+/+*^ and *Xpo4*^*+/−*^ mice. Quantifications are shown (DAPI, blue; R-loop, red). *n* = 6 for both *Xpo4*^*+/+*^ and *Xpo4*^*+/−*^. **b** Representative IF images with enlarged regions of R-loop in *Xpo4*^*+/+*^ and *Xpo4*^*+/−*^ mice. Quantifications are shown (DAPI, blue; R-loop, red). *n* = 6 for both *Xpo4*^*+/+*^ and *Xpo4*^*+/−*^. **c** IF of γH2A.X (DNA damage) in the hippocampus of *Xpo4*^*+/+*^ and *Xpo4*^*+/−*^ mice. Representative images and quantification are shown (DAPI, blue; DNA damage, green). *n* = 6 for both *Xpo4*^*+/+*^ and *Xpo4*^*+/−*^. **d** IF of NeuN, a neuronal marker, in the hippocampus of *Xpo4*^*+/+*^ and *Xpo4*^*+/−*^ mice. Representative images and quantification are shown (DAPI, blue; NeuN, red). *n* = 6 (*Xpo4*^*+/+*^) and 6 (*Xpo4*^*+/−*^). **e** A proposed working model of XPO4 in ecircRNA export and the physiological & pathological relevance. EcircRNAs generated in the nucleus get exported into the cytoplasm, and insufficiency of XPO4 leads to nuclear accumulation of ecircRNAs and then the formation of deleterious ciR-loops that triggers DNA damage and liR-loop buildup. Insufficient XPO4 dosage and ecircRNA export lead to testis and neurological defects in *Xpo4*^*+/−*^ mice. Scale bars, 200 μm. For **a**–**d**, data are shown as means ± SD. *P* values from unpaired two-sided Student’s *t* test and were indicated in the figures. Source data are provided as a Source data file.

The brain is another organ with high ecircRNA abundance^27–29,31^. Next, we assessed the potential neurological relevance of ecircRNA export. Compared to the *wildtype* mice, significant increase of ciR-loop levels was observed in the hippocampus of *Xpo4*^*+/−*^ mice (Fig. 6a). The overall R-loop levels were also significantly increased in the hippocampus of *Xpo4*^*+/−*^ mice (Fig. 6b). The DNA damage levels demonstrated consistent increases in the hippocampus of *Xpo4*^*+/−*^ mice (Fig. 6c). We observed a significant decrease in neuron numbers in the hippocampus of *Xpo4*^*+/−*^ mice compared to *Xpo4*^*+/+*^ mice (Fig. 6d). Correlated with the hippocampal neuronal loss, assays with young adult mice to test hippocampus-dependent working memory and spatial reference memory showed behavioral defects in *Xpo4*^*+/−*^ mice^65–68^ (Supplementary Fig. 7f). Compared to *Xpo4*^*+/+*^ controls, the *Xpo4*^*+/−*^ mice displayed significantly reduced spontaneous alternation in the Y-Maze and significantly longer latency to find the target hole in the training phases of Day 2 in the Barnes Maze (Supplementary Fig. 7g). Collectively, these data implied that sufficient XPO4 and effective ecircRNA export were essential to hippocampal neurons and that in the deficiency of ecircRNA export, nuclear pileup of ecircRNAs would cause deteriorative accumulation of ciR-loops and R-loops and lead to DNA damage and neuronal loss.

## Discussion

This study provided lines of evidence to support a role of the conserved XPO4 in the nuclear export of ecircRNAs in metazoans, and to uncover the abnormalities as nuclear accumulation of a subset of circRNAs, ciR-loop, and R-loop formation, and DNA damage under the condition of XPO4 deficiency (Fig. 6e). At the organismal level, lower XPO4 dosage led to male infertility and neurological defects in *Xpo4*^*+/−*^ mice, and cells in testes and hippocampal neurons of the heterozygotes exhibited the same abnormalities as were observed in cultured XPO4 KO cells (Fig. 6e). Efficient ecircRNA nuclear export is a previously unappreciated layer of regulation that is essential to normal physiology, and abnormalities in it lead to physiological defects. One concern about physiological roles of multi-functional factors such as XPO4 is that it may display biological effects not just through a particular mechanism. Even we have examined and largely excluded the involvements of several known cargos of XPO4 such as eIF5A and Smad3, in the cellular phenotypes of XPO4 deficiency as nuclear accumulation of ecircRNAs and eventually DNA damage, potential contribution of roles of XPO4 other than ecircRNA export in the overall physiological effects of XPO4 deficiency can be further considered. At the whole organism level, possible interference of other XPO4 roles could also be related to the phenotypes demonstrated in the XPO4 heterozygotes. XPO4 could also participate in nuclear export or regulate the cytoplasmic and nuclear distribution of ecircRNAs through other alternative mechanisms. These could include but are not limited to the potential roles of XPO4 in the distribution of proteins that are involved in the metabolism of circRNAs, or proteins that mediate the formation or resolving of ciR-loops. XPO4 could also act in concert with other transport receptors of nuclear RNAs (e.g., Nxf1 and DDX39a/b) to regulate ecircRNA export. These possible direct or indirect roles of XPO4 in regulating circRNA are not exclusive to each other.

Some RNAs need to be kept in the nucleus^4,69,70^, whereas others need to be exported to the cytoplasm^11–14^. Many ecircRNAs function in the cytoplasm; for example, the ecircRNA CDR1as plays role in the mammalian brain as a cytoplasmic competing endogenous RNA (ceRNA) against microRNAs, and circBoule RNAs have conserved roles in animal male fertility as cytoplasmic scaffolds for the degradation of heat-shock proteins^32,71,72^. Although this study concentrated on nuclear abnormalities, the additive effects from both nuclear deteriorative ciR-loops and the absence of cytoplasmic roles of some ecircRNAs may underlie the overall consequences of deficient ecircRNA export. Successful export of both coding and noncoding RNAs is critical in multiple biological processes^11^. Here, we identified the conserved exportin XPO4 as an essential nuclear export of a subset of ecircRNAs in metazoans, and the export of ecircRNAs may be actively regulated to preclude physiological abnormalities that otherwise would be triggered.

XPO4 has been identified as a tumor suppressor in liver cancer and breast cancer, and its role in the export of eIF5A is known to be critical to the tumor-suppressing effects^73^. XPO4’s role in ecircRNA export, which remains to be elucidated in carcinogenesis, has the potential oncogenic relevance since deleterious R-loop formation is a recognized trigger of genome instability, which is a hallmark of cancer^74^. The splicing machinery is extensively involved in normal physiology and human diseases, as previously demonstrated by regulating alternative splicing or causing splicing abnormalities of mRNAs^75^. Our study showed that insufficient canonical splicing can cause nuclear accumulation of ciR-loops and DNA damage and identified a novel component of the disease link of splicing machinery. Eukaryotic cells utilize an array of mechanisms to prevent harmful R-loop formation^44,46,52,54,63,76,77^. DDX39 can resolve ciR-loops and R-loops, and, thus, may also participate in the pathology of related diseases. XPO4 activity, ecircRNA export, and ciR-loop formation must be accurately regulated for normal physiology, and it is highly possible that these components are also involved in an array of other diseases.

Our results reveal conserved roles of XPO4 in the nuclear export of ecircRNAs, and demonstrate that efficient nuclear export of ecircRNAs is essential to animal and human cells. The previously unrealized deleterious ciR-loop formation and the downstream events due to insufficiency in ecircRNA export may be a hidden layer underneath particular physiological abnormalities.

## Methods

### Mice

XPO4^+/−^ mice were generated using the CRISPR/Cas9 system with Cas9 mRNA and sgRNAs were microinjected into fertilized embryos of C57BL/6J mice. All mice were genotyped 2 weeks after birth. Deletions in XPO4 were confirmed by Sanger sequencing analysis. Specific primers for PCR were listed in Supplementary Table 1. All XPO4^+/−^ mice used for analyses were in parallel with age- and gender-matched wild-type littermates as a control group. Mice were housed in groups of five and were given one week to habituate before the start of the experiment upon arrival. Mice were kept in an enriched environment under the standard conditions (22 ± 2°C temperature, 40–60% humidity) with a 12-h light/dark cycle (lights on from 07:00 to 19:00) at a stable temperature (23–25 °C) at the Specific-Pathogen-Free (SPF) facility. Mice were randomly assigned to experimental groups. Sample size was based on calculation and reported publications. All animal protocols were approved by the Animal Care and Use Committee of the University of Science and Technology of China (USTCACUC192001039).

### Cells and cell culture

Human and mouse cell lines including HEK293T (human fetus origin) and NIH/3T3 cells (mouse embryo origin) were purchased from the American Type Culture Collection (ATCC; http://www.atcc.org). NIH/3T3 XPO4 KO cells were generated using CRISPR-Cas9. Both copies of the XPO4 gene are edited in the exact same manner. Knockout of XPO4 was confirmed by Sanger sequencing analyses. Specific primers for PCR were listed in Supplementary Table 1. All human and mouse cells were cultured with DMEM under standard conditions including 10% FBS and 1% penicillin/streptomycin at 37 °C under 5% CO_2_. For FLAG-tagged Ranbp16 stable Drosophila S2 cells, S2 cells were transfected with Hy-pAct5c-FLAG-Ranbp16 for 48 h, and then maintained by selection with 150 μg/mL hygromycin B for 3 weeks. S2 cells were maintained at 25 °C using Schneider’s Drosophila medium (SDM, Sigma, S9895) with 10% FBS (HyClone, SH30910.03) and 1% (v/v) penicillin-streptomycin (Thermo Fisher Scientific, 15140122). Cells were tested for mycoplasma by DAPI staining, to ensure the absence of contamination.

### Construction of plasmids

All plasmids were constructed with restriction enzyme digestion and ligation, or with a recombination method. For overexpression with Exportins, eIF5A, Smad3, eIF4E, Rpa1, Ddx39a, Ddx39b C-terminal FLAG-tag transfection in this study, sequence of the target was cloned into the p3xflag-myc-cmv-24 between HindIII and BamHI sites. To generate the plasmid expressing FLAG-tagged Ranbp16 (Hy_pAct5C FLAG-Ranbp16), the Ranbp16 CDS was inserted into the MCS site of our previously-described Hy_pAct5C Flag MCS plasmid^78^. The shRNA plasmid for knockdown of eIF5A mRNA (sheIF5A, TRCN0000010985), RPA1 mRNA (shRPA1, TRCN0000071282), SRSF1 mRNA (shSRSF1, TRCN0000109143), SF3A1 mRNA (shSF3A1, TRCN0000109205), SF3B1 mRNA (shSF3B1, TRCN0000123809) with negative-control shRNA (shC002) was obtained from the MISSION shRNA Library (Sigma-Aldrich, Germany). All constructions were sequenced for confirmation. Further information about these plasmids can be obtained upon request.

### Transfection of plasmids, dsRNAs, shRNAs

For plasmid, shRNA transfection of human and mouse cells, transfections were conducted with Lipofectamine 2000 (Invitrogen) according to the manufacturer’s protocols. For plasmid transfection of Drosophila S2 cells, transfections were conducted with Effectene transfection reagent (QIAGEN, 301425) according to manufacturer’s protocols. For RNAi in S2 cells, 8 μg of the indicated dsRNA was introduced into 3 × 10^6^ cells. Cells were harvested for further analysis or downstream experiments 72 h post-transfection.

### RNA immunoprecipitation (RIP)

Antibody was incubated with Dynabeads Protein G (Invitrogen) according to the manufacturer’s recommendations. Cells were harvested 48 h post-transfection. Cells were washed twice with PBS and cross-linked with UV (254 nm, 400 mJ/cm^2^, 2 min). RIPA buffer (50 mmol/L Tris-HCl, pH 8.0, 150 mmol/L NaCl, 5 mmol/L EDTA, 1% NP-40, 0.1% SDS) were used to lysis the cells followed by sonication for 10 min and centrifugation at 13,000 × *g* for 15 min at 4 °C. Cell lysates in the supernatant were preserved and then incubated with antibody-coupled beads for the immunoprecipitation procedures. After 4 h incubation at room temperature, beads were washed three times with RIPA buffer and 100 µl of the samples were preserved for Western blot analysis. The rest was subjected to TRIzol RNA extraction followed by standard reverse transcription and quantitative real-time PCR (qPCR). Oligos used are shown in Supplementary Table 1.

### Western blotting

For western blotting, samples were separated on SDS-PAGE gels and then transferred to nitrocellulose membranes (PALL Corporation). Membranes were processed following the ECL Western blotting protocol (GE Healthcare). The information of antibodies is provided in Supplementary Table 1. Images were taken with the ImageQuant LAS4000 Biomolecular Imager (GE Healthcare). For the preparation of the mice brains, brains were isolated and freshly frozen in liquid nitrogen. The tissues were homogenized in RIPA buffer (50 mmol/L Tris-HCl, pH 8.0, 150 mmol/L NaCl, 5 mmol/L EDTA, 1% NP-40, 0.1% SDS, 1 μM dithiothreitol, 1 × protease/1 × phosphatase inhibitor cocktail (Sigma, St. Louis, MO)), followed by the above western blotting

### RNA extraction, reverse transcription, and quantitative PCR

Total RNA was extracted using TRIzol reagent (Invitrogen), according to the manufacturer’s recommendations, and purified by phenol-chloroform extraction after DNase I treatment. The RNA concentration was measured by NanoDrop 2000c (Thermo Fisher Scientific, Life Sciences), and was reverse-transcribed with PrimeScript RT Master Mix for cDNA with the supplied protocol (RR036A, TaKaRa Biosystems). Quantitative PCR (qPCR) was performed with GoTaq SYBR Green qPCR Master Mix (Promega) on a QuantStudio 3 Real-time PCR system (Thermo Scientific, USA) according to standard procedures.

### In vitro transcription, dsRNA producing, and circularization

Templates for in vitro transcription were amplified by primers containing T7 RNA polymerase promoter sequences from the vector containing circADARB1 fragment flanked by group I self-splicing introns of the T4 phage Td gene. circRNA precursor were synthesized by a TranscriptAid T7 High Yield Transcription Kit (Thermo Scientific) and the incubation system was modified as previously described^5^. In vitro transcribed RNAs were extracted by phenol-chloroform (pH ∼ 4.5), precipitated with ethanol and resuspended in RNase-free water. For circularization^79^, the in vitro transcribed RNAs were incubated with T4 RNA ligase I (NEB) at 16 °C overnight according to the manufacturer’s recommendations. Circularized RNAs (and remaining linear RNAs) were then separated on 5% Urea PAGE gel. circRNA bands were excised from the gel and eluted overnight in gel elution buffer (20 mM Tris-HCl, pH 7.5, 250 mM NaOAc, 1 mM EDTA, 0.25% SDS). After elution, the gel fragments were discarded by centrifugation at 15,000 × *g* 4 °C for 2 min. circRNA was then purified using Phenol-chloroform (pH ∼ 4.5) and precipitated with ethanol. dsRNAs were produced by in vitro transcription using ScriptMAX Thermo T7 Transcription Kit (TOYOBO, TSK101). DNA templates were produced by PCR using primers containing the T7 promoter sequence. Details for these primers, dsRNAs are provided in Supplementary Table 1.

### Protein expression and purification

The full-length XPO4, XPO5, and Ran were codon optimized and cloned into the pET-28a vector containing an N-terminal 6 × His-SUMO-TEV tag using restriction sites Nde I and Xhol I, respectively. The proteins were expressed in Escherichia coli BL21(DE3) cells. Cells were culture in Luria-Bertani (LB) medium at 37 °C till the OD600 reached 0.6, proteins expression were induced by 0.1 mM isopropyl β-D-1-thiogalactopyranoside (IPTG) for 16 h at 16 °C. Cells were harvested and resuspended using lysis buffer (20 mM Tris, 400 mM NaCl, 5 mM imidazole, pH 7.8). Cells were lysed by sonication and supernatant were isolated by centrifuge. Proteins were purified by loading supernatant onto Ni-NTA Resin column (GE Healthcare), and followed by 10 CV wash using wash buffer (20 mM Tris, 400 mM NaCl, 50 mM imidazole, pH 7.8). Recombinant proteins were then eluted by elution buffer (20 mM Tris, 400 mM NaCl, 300 mM imidazole, pH 7.8). Eluted proteins were dialyzed and TEV protease-digested in dialysis buffer (20 mM Tris, 150 mM NaCl, 1 mM EDTA, pH 7.4) overnight at 4 °C. Gel filtrations were performed to purify XPO4, XPO5, and Ran by using ÄKTA pure system connecting a superdex S200 16/600 column (Cytiva). Fractions corresponding to XPO4, XPO5, and Ran were collected and concentrated to 10 mg/ml in storage buffer (20 mM Tris, 150 mM NaCl, 20% v/v glycerol, pH 7.4) and stored at −80 °C, respectively.

### Gel shift (EMSA) and supershift

circRNAs were pre-treated as follows. RNAs were incubated at 90 °C for 3 min, then cooled at room temperature for 10 min. MgCl_2_ and Na-HEPES (10 mM MgCl_2_, 50 mM Na-HEPES, pH 8.0) were added and the solution was incubated at 50 °C for 20 min, then cooled at room temperature for 10 min. All of the RNA-protein incubation steps were performed at 4 °C. XPO4 and the indicated proteins were mixed with 4 μmol folded circADARB1 at a molar ratio of 1:1, 2:1, 4:1, and 10:1 in incubation buffer (20 mM Tris, 150 mM NaCl) and incubated for 45 min. Formaldehyde was added for cross-linking with the final concentration of 1% for 15 min. For super-shift assays, Ran and its mutant were added to the pre-incubated mixture and incubate the solution for additional 30 min on ice. The samples were subsequently loaded onto a 6% native TBE gel. Electrophoresis was performed at a constant voltage of 100 V for 3 h. The resulting gels were visualized by GelRed staining.

### Nucleocytoplasmic separation

Cells were trypsinized and resuspend with slow pipetting in 1 mL of Lysis Buffer B (10 mM Tris-HCl pH 8.4, 140 mM NaCl, 1.5 mM MgCl_2_, 0.5% NP-40). Spin 1000 × *g* at 4 degrees for 3 min. The supernatant was kept as cytoplasmic fraction. For nuclear fraction, resuspend the pellet in 1 mL of Lysis Buffer B in addition with 1/10th volume (100 μL) of Detergent stock (3.3% (w/v) Sodium Deoxycholate, 6.6% (v/v) Tween 40) under slow vortex, and keep it on ice for 15 min. Spin 1000 × *g* at 4 degrees for 3 min and wash the pellet with Lysis Buffer B twice before adding 1 mL of TRIzol for standard RNA extraction.

### Library construction, high-throughput sequencing, and the corresponding bioinformatics analyses

The RNAs from RIP sample or nucleocytoplasmic separation sample were utilized to construct whole transcriptome library by the TruSeq Ribo Profile Library Prep Kit (Illumina, United States), following manufacturer’s instructions. For circRNA sequencing, linear RNA was digested with 3 U of RNase R (Epicentre, USA) per μg of RNA before library construction. In brief, rRNA was removed by Illumina Ribo-Zero Gold kit and surplus RNA was purified for end repair and 50-adapter ligation. Then, reverse transcription was performed with random primers containing 3′ adapter sequences. Finally, the cDNAs were purified and amplified with PCR reaction. The products with 300–500 length were purified and quantified. These libraries were utilized to 150 nt paired-end sequencing with an Illumina Nova seq 6000 system (Novogene, China). Each library was generated a depth of ~8 G basepairs (for nucleocytoplasmic separation sample) or ~6 G basepairs (for RIP sample), and then adapters were removed with cutadapt software to obtain cleandata. In order to analysis the cleandata of circRNAs sequencing in RIP-seq data or nucleocytoplasmic separation sequencing data, the find_circ software (v 1.2) was utilized to predict the junction of circRNAs. The junction reads were normalized to transcripts per million (TPM). The same circRNAs detected from two replicates were merged using R script. To determine the significance, Student’s *t* tests were used to calculate *P*-values in R software. To analyze the differences of circRNA expression level between nuclear and cytoplasm fraction, the DEseq2 (v 1.28.1) package was used to calculate Nuc/Cyto fold changes of circRNA in WT and XPO4 KO cell, respectively. The circRNAs significantly enriched (mandatorily defined) in the cytoplasm (log2(Nuc/Cyto) <−0.3, *P* < 0.05) or nuclear fraction (log2(Nuc/Cyto) >0.3, *P* < 0.05) were utilized to analyze the length distribution, expression level, G/C content and ΔG distributions. The circRNA length and G/C content were calculated in R software based on the circBase (http://www.circbase.org) data. The ΔG was computed using the RNAfold program of the ViennaRNA package with default parameters. For data visualization, the boxplots and cumulative fraction curves were generated by ggplot2 (v 3.3.3) package in R software. The circos plots were generated by circlize packge (v 0.4.13) in R software. The heatmaps and metaplots of circRNA levels in RIP-seq were generated by deeptools. The percentage of circRNA reads were calculated by samtools.

For analysis of EIciRNAs, reads were aligned to the grcm38 genome with bwa software (v 0.7.17) and back-splicing junctions were determined by CIRI2. To decide whether these circRNAs include any introns, the pair-end reads for back-splicing junction reads were found. CircRNAs with any intronic sequence on these pair-end reads were defined as EIciRNAs.

### DNA–RNA hybrid immunoprecipitation (DRIP-seq) and the corresponding bioinformatics analyses

Totally 10^7^ cells were trypsinized and centrifuged for the cell pellets. Cells were resuspended by 4 ml TE buffer with 0.5% SDS and 0.1 mg/ml proteinase K, incubated in constant temperature shaker at 37 °C for 16 h. Add 1/4 volume 5 M KAc, mix and place the tube on ice 10 min. Then the DNA was extracted by phenol: chloroform: isoamyl alcohol (25:24:1). Precipitate the supernatant by adding 1 volume isopropyl alcohol, 30 min at room temperature, and washed by 1 ml 70% ethanol. Dry the pellet and resuspend by 200 μl ddH_2_O. A cocktail of restrict enzymes (MseI, DdeI, AluI, MboI; NEB) was used for gDNA digestion in 200 μl system, incubated at 37 °C overnight. Fragmented DNA was purified and quantified by Qubit 3.0 (Invitrogen). Mix 1.8 µg DNA with 0.2 µg *E. coli* DNA (for normalization) in 500 μl 1 × TE. For RNase R treated group, 10 U RNase R (Epicentre) incubated at 37 °C for 1.5 h was used to remove linear RNA from DNA Fragment. For control group, the identical amount of reaction buffer was added to DNA Fragment and incubated at 37 °C for 1.5 h. Take out 50 μl as Input, the rest 450 μl DNA was immunoprecipitated overnight at 4 °C with 1× DRIP binding buffer (10 mM NaPO_4_ pH 7.0, 140 mM NaCl, 0.05% Triton X-100) and 10 µg S9.6 antibody (ATCC, HB-8730) at 500 μl system. Add 50 µl Protein G Dynabeads (Invitrogen, 10004D) and incubated for 4 h, then wash the beads by 1× DRIP binding buffer for 4 times at room temperature. Add 250 µl elution buffer (50 mM Tris pH 8.0, 10 mM EDTA, 0.5% SDS) and 5 U Proteinase K to the bead-antibody complexes, and incubated for 60 min in an Eppendorf ThermoMixer at 55 °C.

DNA from DRIP was purified and subjected to library construction using Accel-NGS® 1S Plus DNA Library Kit (Swift Biosciences). These libraries were utilized to 150 nt paired-end sequencing with an Illumina Nova PE150 system (Genewiz, China). Each library was generated from 70 to 121 million reads and then adapters were removed with cutadapt software to obtain cleandata.

Reads from cleandata were aligned to the grcm38 genome with Bowtie2 version 2.4.1 using default settings, with all duplicates, unmapped reads, reads with more than three mismatches and non-uniquely mapped reads were removed by using samtools version 1.1. MACS2 was used to identify peaks in individual replicates (-q 0.001). The bamCoverage pipeline in deeptools (v 3.5.1) were used to generate R-loop reads coverage files (.bw). The .bw files from two replicates in each group were merged by bigwigCompare pipeline (-operation mean) for IGV visualization. The common peaks from the replicates were merged using bedtools (v2.30.0). The coverage matrix of each group was calculated with computeMatrix and the heatmap and Metaplot was generated with plotHeatmap and plotProfile pipeline in deeptools, respectively. The peaks from RNase R treated group was regarded as ciR-loops.

ciR-loops from genomic regions that could generate circRNAs were defined as “*cis*” ciR-loops. On the other hand, ciR-loops from genomic regions that could not generate circRNAs were defined as “*trans*” ciR-loops. It is worth noting that DRIP-seq actually sequenced the DNA strands rather than RNA strands of the R-loops, which would underestimate ciR-loops formed in trans, and at the same time overestimate cis ciR-loops, because ciR-loops detected at a genomic locus that gives rise to a circRNA would be counted as *cis* but not as *trans* ciR-loops, even they might be actually formed “in trans” by circRNAs encoded by the other genomic loci.

For liR-loops, two subtypes were grouped either formed at genomic loci with known annotated mouse transcripts (WAT) or without known annotated mouse transcripts (WOT). Annotated mouse transcripts were based on: www.gencodegenes.org. WAT liR-loops would be overestimated and WOT liR-loops would be underestimated, for the same reason stated above for *cis* and *trans* ciR-loops.

The correlation coefficient and *P*-value of ciR-loop levels between ciR-loop and liR-loop levels was calculated in R software, and the scatterplot was generated by ggplot2. Metaplots, boxplots, and cumulative fraction curves were generated with deepTools and R scripts.

Total RNAs of 3T3 cells were subjected to RNA-sequencing, and the RNA-seq data were used to calculate the correlation between gene expression and R-loops. Hisat2 and feature counts software were used to map clean reads from the RNA-seq data to reference genome (grcm38), and the gene expression level was normalized to FPKM.

### Immunofluorescence (IF) staining

Slides with the corresponding human and mouse cells were incubated with antibody against S9.6 (Kerafast, ENH001, 1:200), γH2A.X (CST, 2577S, 1:200) overnight at 4 °C followed by incubation with fluorescence secondary antibody for 2 h. Images were taken through confocal microscopy (Olympus IX-71). The intensity of the nuclear fluorescence signals was analyzed using Image J software. For S2 cells, coverslips used for S2 cells were coated with concanavalin A (Con A; Solarbio, C8110) to promote attachment before use. After RNAi, 0.5 × 10^5^ cells were seeded onto a ConA-coated coverslip in a well of the 6-well plate for 2 h. Coverslips (cell side up) were incubated with an antibody against S9.6 (Kerafast, ENH001, 1:200) for 12 h at 4 °C followed by incubation with fluorescence secondary antibody at room temperature for 2 h (Abcam, ab150116, 1:100). Fluorescence signals were captured with a Leica confocal system (TCS SP8 DIVE). The intensity of the nuclear fluorescence signal was analyzed using Image J software. For mice brains, 4-month-old mice were anesthetized by isoflurane and transcardially perfused with ice-cold phosphate buffer (PBS). The brains were isolated and fixed in 4% paraformaldehyde (PFA) at 4 °C for 24 h and then kept in 30% sucrose dissolved by phosphate-buffered saline (PBS) at 4 °C until brains sank. Following dehydration, brains were sectioned to 35 μm. Immunofluorescence staining was performed after washing slices three times with PBS, permeabilizing slices with 0.5% Triton X-100 in PBS for 15 min, and blocking slices with 3% normal goat serum and 0.1% Triton X-100 in PBS for 1 h at room temperature. The mouse brain slices were incubated with the primary antibodies against S9.6 (Kerafast, ENH001, 1:200), γH2A.X (CST, 2577S, 1:200), NeuN (EMD Millipore, MAB377, 1:500) applied in 3% normal goat serum and 0.1% Triton X-100 in PBS followed by incubation of secondary antibody. Brain images of mice samples were taken through an upright microscope (Axio Imager Z2, Carl Zeiss, Germany) and Tissue FAXS 6.0 software from Tissue Gnostics; an inverted laser scanning confocal microscope (LSM 800, Carl Zeiss, Germany) and ZEN software from Zeiss. The intensity of the nuclear fluorescence signal was analyzed using Image J software. For UV treatment, NIH/3T3 wildtype cells were treated with UV (254 nm, 1 J/cm^2^, 3 min) in UVP HL-2000. For RNase H treatment, slides were incubated with RNase H (ThermoFisher, 18021014, 3 U/μg) for 1.5 h before primary antibody incubation. For RNase R treatment, slides were incubated with RNase R (Epicentre Biotechnologies, RNR07250, 3 U/μg) for 1.5 h before primary antibody incubation.

### Fluorescence in situ hybridization (FISH)

Fluorescence in situ hybridization was performed as previously described^5^. The TranscriptAid T7 High Yield Transcription Kit (Thermo Scientific) was used to transcribe RNA probes spanning the backsplicing junction of circRNAs. RNA probes were labeled by ULYSIS Nucleic Acid Labeling Kit (Invitrogen) with Alexa Fluor 546 Fluorescent dyes to specifically detect circRNAs. DNA probes targeting introns were prepared by sonicating amplicons of genomic PCR and then labeled with a ULYSIS Nucleic Acid Labeling Kit (Invitrogen) with Alexa Fluor 488 Fluorescent dyes to specifically detect DNA loci of encoded circRNAs. Cells were fixed by fixative (methanol:acetic acid = 3:1) and dehydrated with gradient ethanol (70%–90%–100%). The fixed cells and probes were denatured at 80 °C for 10 min and then incubated at 37 °C overnight. Cell slides were washed with 2×SSC + 0.1% Triton X-100 at 55 °C for 1 h. For IF-FISH, FISH assay was immediately performed after IF.

### Quantification of FISH and IF images

For all FISH and IF experiments, imaging conditions were set in parallel, with identical exposure times and laser settings. Images were analyzed and quantified using ImageJ. Statistical analysis and data visualization were conducted using GraphPad or R software. *P* values were determined by Student’s *t* test. In detail, nuclear or total cell mean signals were determined from a single plane for individual images. Nuclear regions were defined by thresholding or manual tracing DAPI. For circRNAs FISH quantitation, whole cell and nuclear signals were both quantified for further analysis. For S9.6 quantitation, only nuclear S9.6 signals were used for R-loop quantitation, with all cytoplasmic staining was excluded before analysis. Images were analyzed using ImageJ that measured S9.6 staining in nuclei only based on DAPI staining. In order to minimize staining-based sample deviation, the mean value for each individual experiment condition was standardized to the negative control.

### Comet assay

Comet assays were conducted using Comet SCGE assay kit (Enzo, ADI-900-166). Cells were washed with Ca^2+^/Mg^2+^ free 1 × PBS (Biosharp, BL551A) and combined gently with molten LMAgarose (1:10 dilution; LMAgarose was boiled for 5 min and incubated at 37 °C for 20 min before use). Mixture was immediately pipetted onto a Comet Slide. Slides (cell side up) were placed at 4 °C in the dark for 30 min, immersed in pre-cold Lysis Solution at 4 °C for 40 min, and treated with freshly-prepared alkaline solution (300 mM NaOH, 1 mM EDTA, pH >13) at room temperature in the dark for 40 min. Then Slides were washed with 1×TBE buffer (89 mM Tris base, 89 mM boric acid, 2.5 mM EDTA) twice, electrophoresed in 1×TBE buffer for 10 min (1 volt per cm of distance between positive and negative electrodes), dehydrated in 70% ethanol for 5 min, and dried at room temperature. 100 μl of CYGREEN Dye (dissolved in Deionized Water; 1:1000 dilution) was added to each slide for 30 min. Slides were washed with 1 × TBE buffer and dried completely at 37 °C in the dark. Images were acquired by a Leica confocal system (TCS SP8 DIVE). DSB was examined by measuring tail length using CASP software (http://casplab.com/).

### Cell synchronization (G2/M)

HEK293T cells were added 5 mM thymine (Sigma) for 16 h. Then the cells were washed 3 times with fresh DMEM media, and added 25 μM 2′-Deoxycytidine (Sigma) to release for 8 h. Repeat the above steps before harvesting the cells. Cell stages were confirmed by FACS.

### *C. elegans* methods

For *C. elegans* culture and strains, all strains were maintained on nematode growth media (NGM) seeded OP50 at 20 °C. N2 Bristol was obtained from the Caenorhabditis Genetic Center (CGC).

For plasmid construction, the plasmid pDD162 that expresses Cas9 II protein was used^80^. Four sgRNAs were designed to target Y69A2AR.16, two of which target exon14 and the others target exon16. We selected sgRNAs containing two NGG PAM motif in the 3′ ends of sgRNA sequences to increase the efficiency of the sgRNAs. We used http://crispor.tefor.net/ to assess the usability of sgRNAs. To generate the plasmid expressing sgRNAs, the 20 nt sgRNA sequence was inserted between the EcoRI and HindIII restriction endonuclease sites at the 3′ end of the U6 promoter.

For injection of CRISPR/Cas9 knock-out and knock-in and other plasmids, we carried out CRISPR/Cas9 system as previously described with modification^81^. We mixed 4 plasmids expressing sgRNAs (100 ng/μL of each), pDD162 (100 ng/μL) expressing Cas9 II protein, and the plasmid (PCFJ90) (100 ng/μL) expressing mCherry in the pharynx together. The mixture was injected into about 30 N2 adults (adulthood day 1).

For RNA extraction, reverse transcription, and qPCR, worms were collected at young-adult stage by washing 4 times with M9 buffer, and were pelleted by centrifugating at 600 × *g* for 1 min. Total RNA was extracted by using TRIzol L/S (Invitrogen).

For worm synchronization, gravid adult worms were collected into 1.5 mL tubes by washing with M9 buffer. Then we washed the worms 3 more times and collect the worms by centrifuging at 600 × *g*. Worms were treated with hypochlorite, and synchronized embryos were cultured at 20 °C on NGM plates.

For brood size counts, worms were synchronized for 3 times. 10 adult worms were allowed to lay eggs for 2 h at 20 °C and then the worms were removed. Eggs were allowed to hatch and grow until L4 stage. 22 worms were plated on 35 mm NGM plates, respectively. The worms were transferred to another NGM plate every day. The number of living offspring was calculated after no eggs were laid.

For examining of development stages, worms were synchronized for 3 times. 10 adult worms were allowed to lay eggs for 1 h at 20 °C, and then the worms were removed. The eggs were allowed to hatch and grow on NGM plates with adequate food. The development stages were recorded after 30 h, 42 h, 54 h, and 66 h after the eggs were laid.

For cytological preparation and immunostaining, synchronized worms were collected and washed by M9 buffer for 3 times and egg buffer twice. Supernatant was removed and 100 μl egg buffer was left. Then levamisole was added to narcotize the worms, and the worms were dissected for germlines by removing the head and tail using a fine gauge needle (27 G). The germlines were transferred to on a poly-L-lysine coated slide (slides were washed in 70% ethanol and 1% HCl, then given 1 coat of 100% poly-L-lysine, air dried). 100 μl 4% paraformaldehyde (PFA) in PBS was added on the germlines, and the slides were placed in a humid chamber. After 15 min, the PFA were removed and the germlines were freeze-cracked. The slides were soaked in methanol pre-cooled in −20 °C for 5 min. The slides were washed in PBST (PBS + 0.5% Triton X-100) for 3 × 10 min. Then the samples were blocked for 1 h by adding 100 μl PBST (PBS + 0.1% Tween-20 with 1% BSA) on the germlines in the humid chamber. Primary antibody was diluted in PBST (1:200) and incubated overnight at 4 °C in the humid chamber. The slides were then washed in PBST for 3 × 5 min before incubation with the secondary antibody (1:500) for 4 h at room temperature. Finally, germlines were washed at least 3 × 5 min in PBST before mounting with a coverslip on DAPI with mounting medium.

### Sperm morphology and CASA assay

The semen samples from 6 adult male mice in each group were obtained from the cauda epididymis and diluted for 15 min at 37 °C with 1 ml capacitation solution. Sperm morphology was analyzed by hematoxylin and eosin (H&E), and observed through microscopy system. The parameters (percentage of progressive cells and percentage of motile cells) of semen were further analyzed by a computer-assisted analysis system.

### TUNEL staining

Testis issues were fixed with 10% (wt/vol) formalin followed by standard paraffin section preparations. The slides were then stained with the In Situ Cell Death Detection Kit, Fluorescein (Roche, 11684795910), following the manufacturer’s instructions. Images were taken through confocal microscopy (Olympus IX-71). Apoptotic signals were quantified by counting the number of cells positive for TUNEL staining within each seminiferous tubule. Images were analyzed using ImageJ with thresholding. In order to minimize staining-based sample deviation and unspecific labeling, the mean value for each individual experiment condition was standardized to the negative control.

### Mice behavioral experiments

Y-maze is used to detect animal’s hippocampal-dependent working memory, which consists of three same arms separated by 120°. Briefly, mice were placed in the initial arm of the device and allowed to explore freely, and the order of the mice entering different arms was recorded for 5 min. The number of entry time was considered as an index of locomotion, and the ratio of time in the discontinuous three arms to total arm, spontaneous alteration, was considered as an index of working memory.

Barnes maze is used to detect animal’s spatial reference memory, which consists of a circular platform with 20 holes distribution around the periphery. One of the holes contains an escape box where mice can hide. There are four headlights on the platform used to drive mice to avoid intense light stimulation. Before experiment, the mice adapted in the dark for 1 min in a black box, then open the box and were allowed to escape freely. Mice were trained for 4 days, 4 trials daily, each training period is 180 s. At the interval of each trial, the platform was cleaned with 75% alcohol. The latency of the mice to find the target hole was recorded and considered as an index of spatial reference memory.

### Quantification and statistical analysis

Information on specific quantification methods is described in associated Method details, or main texts. High-throughput sequencing data were analyzed in Method details. Statistical tests were performed using R software. The statistical significance was evaluated by Student’s *t* test for data which follow the normal distribution. For others, non-parametric tests such as Wilcox rank-sum test or Mann–Whitney U test were utilized to evaluate the statistical significance as indicated in the figure legends. All statistical graphs were generated by GraphPad Prism (v. 8.0.2) or ggplot2 in R software, with error bars showing SD. Parameters such as number of sample size, the number of mice, the number of independent experiments, statistical test and significance, are reported in Figures and Figure Legends or Method details.

For data process of RIP-qPCR and nucleocytoplasmic separation-qPCR in Figs. 1a, 1b, and 1e, the enrichment was defined as the relative fold change between antibody and IgG (or nuclear and cytoplasm) which was calculated by ΔCt value. The P values were computed by unpaired two-sided Student’s *t* test with the relative fold change from three independent experiments.

For statistical analysis of FISH and IF fluorescent signals (Figs. 2h–m and 3, Supplementary Fig. 1l; Supplementary Fig. 3a–h, j; Supplementary Fig. 4a, d, g, h), mean nuclear and total signals were obtained as the fluorescence intensity per area for further analysis. The intensity or nuclear/total ratio was normalized to the corresponding control (labeled in boxplots with mean relative control signal = 1). *P* values were from unpaired Student’s *t* test, and were calculated by relative value of the intensity or nuclear/total ratio. Every cells were randomly selected for statistical analysis.

### Reporting summary

Further information on research design is available in the Nature Research Reporting Summary linked to this article.

## Supplementary information

Supplementary Information

Reporting Summary

## Data Availability

The RNA-seq data generated in this study have been deposited in the GEO database under accession code GSE196317. The DRIP-seq data generated in this study have been deposited in the GEO database under accession code GSE196318. The processed high-throughput data are available at GEO database in same accession code. The statistics data generated in this study are provided in the Source data file. Source data are provided with this paper.

## Source data

Source Data
