# Supplementary material for: Exportin 4 depletion leads to nuclear accumulation of a subset of circular RNAs
Source: Nat Commun. 2022 Oct 1;13:5769. doi: 10.1038/s41467-022-33356-z (PMC9526749; doi:10.1038/s41467-022-33356-z)

Supplementary Figure Legends

Supplementary Fig. 1 | XPO4 in nuclear export of ecircRNAs. a, Phylogenetic tree of XPO4 homologs across species. **b**, Evaluation for the purity of the cytosolic and nuclear fractions after nucleocytoplasmic separation with *Drosophila* S2 cells with reverse transcription quantitative PCR (RT-qPCR) of several RNAs. **c**, Heatmap representation of relative circRNA binding of endogenous exportins from RNA immunoprecipitation (RIP) in human HEK293T cells. RT-qPCR of individual ecircRNA was performed, and the enrichment was normalized to the immunoglobulin G (IgG) control. **d,** Relative enrichment of several linear RNAs in XPO4 endogenous RIP. The enrichment was normalized to the immunoglobulin G (IgG) control. **e**, Number and genomic distribution of ecircRNAs in the RIP-seq of endogenous XPO4 (IgG as the negative control). **f, g,** Representative FISH images of ecircRNAs (red) upon siRNA knockdown in human HEK293T cells and mouse NIH/3T3 cells. siCtrl, siRNA negative control with scrambled sequences. Scale bars, 20 μm. n=number of cells analyzed. **h**, Evaluation for the purity of the cytosolic and nuclear fractions after nucleocytoplasmic separation in WT and XPO4 KO cells with RT-qPCR of several RNAs. **i**, Levels of EIciRNAs in the nucleus of 3T3 WT and XPO4 KO cells. n=number of EIciRNAs analyzed. **j,** GC content distributions of WT and XPO4 KO cells enriched in the cytoplasmic fraction ($\log_2(Nuc/Cyto) < -0.3$). **k,** ΔG distributions of WT and XPO4 KO cells enriched in the nuclear fraction ($\log_2(Nuc/Cyto) > 0.3$). **l**, Representative FISH images of ecircRNA (red) in HEK293T cells with mixed stages or in cells at the G2/M stage (synchronized cells, before the breakdown of nuclear envelope). Quantitation of relative FISH signals in the nuclei is shown; n = number of cells analyzed. Scale bars, 20 μm. For **f-g**, **i**, **l**, boxes for box-plot graphs extend from the 25th to 75th percentiles and the line in the middle is plotted at the mean. Whiskers delineate all data points from minimum to maximum. For **b, d, f-i, l**, data are shown as means ± SD. For **b**, **d**, **h**, data are from three independent experiments. For **c, d, f-g**, **i-l**, P values were from unpaired two-sided Student's *t* test and were indicated in the figures, ns: not significant. Source data are provided as a Source Data file.

Supplementary Fig. 2 | Examining the relevance of eIF5A, Smad3 and eIF4E to ecircRNA distribution changes. a, shRNA knockdown (KD) efficiency of eIF5A mRNA with RT-qPCR in WT and XPO4 KO cells. Short-hairpin construct (sheIF5A)

is used for the knocking down. **b**, RT-qPCR of individual ecircRNA for the nuclear and cytoplasmic distribution upon eIF5A KD in 3T3 WT or XPO4 KO cells. **c**, Validation of eIF5A over-expression (OE) in WT and XPO4 KO cells with RT-qPCR. **d**, RT-qPCR of individual ecircRNA for the nuclear and cytoplasmic distribution upon eIF5A OE in 3T3 WT or XPO4 KO cells. **e**, siRNA knockdown (KD) efficiency of Smad3 mRNA with RT-qPCR in WT and XPO4 KO cells. Small interfering RNA (siSmad3) is used for the knocking down. **f**, RT-qPCR of individual ecircRNA for the nuclear and cytoplasmic distribution upon Smad3 KD in 3T3 WT or XPO4 KO cells. **g**, Validation of Smad3 over-expression (OE) in WT and XPO4 KO cells with RT-qPCR. **h**, RT-qPCR of individual ecircRNA for the nuclear and cytoplasmic distribution upon Smad3 OE in 3T3 WT or XPO4 KO cells. **i**, siRNA knockdown (KD) efficiency of eIF4E mRNA with RT-qPCR in WT and XPO4 KO cells. Small interfering RNA (sieIF4E) is used for the knocking down. **j**, RT-qPCR of individual ecircRNA for the nuclear and cytoplasmic distribution upon eIF4E KD in 3T3 WT or XPO4 KO cells. **k**, Validation of eIF4E over-expression (OE) in WT and XPO4 KO cells with RT-qPCR. **l**, RT-qPCR of individual ecircRNA for the nuclear and cytoplasmic distribution upon eIF4E OE in 3T3 WT or XPO4 KO cells. In the corresponding experiments, EV, empty vector used to overexpress the corresponding protein; siCtrl, siRNA negative control with scrambled sequences; shCtrl, the vector used to express the short-hairpin construct for knocking down the expression of the corresponding protein gene. For **b, d, f, h, j, l**, Gapdh mRNA and U6 snRNA are cytoplasm and nucleus enriched RNA, respectively, and are used as references for effective separation of cytoplasm and nuclei. For **a-l**, data are from three independent experiments. P values from unpaired two-sided Student's *t* test. Data are shown as means ± SD. P values were indicated in the figures, ns: not significant. Source data are provided as a Source Data file.

Supplementary Fig. 3 | Examining the relevance of eIF5A, Smad3 and eIF4E to the cellular phenotypes of XPO4 deficiency and effects of XPO4 deficiency in *Drosophila* cells. **a**, IF of R-loops and γH2A.X (DNA damage) upon eIF5A overexpression (OE) in XPO4 KO cells. **b**, IF of R-loops and γH2A.X upon eIF5A KD (sheIF5A) in 3T3 cells. **c**, IF of R-loops and γH2A.X upon Smad3 overexpression (OE) in XPO4 KO cells. **d**, IF of R-loops and γH2A.X upon Smad3 KD (siSmad3) in 3T3 cells. **e**, IF of R-loops and γH2A.X upon eIF4E KD (sieIF4E) in 3T3 cells. **f**, IF of R-loops and γH2A.X upon eIF4E overexpression (OE) in XPO4 KO cells. **i**, IF of R-loops

with or without the RNase H digestion in *DmXPO4* knockdown S2 cells. Representative images and quantification are shown (R-loop, red). Knockdown was achieved with *DmXPO4* dsRNA, and β-gal dsRNA was used as a negative control. **j**, IF of R-loop without or with (ciR-loop) the RNase R digestion in *DmXPO4* knockdown S2 cells. Representative images and quantification are shown (R-loop, red). **k**, Comet assay upon knockdown of *DmXPO4* with S2 cells. Representative comet assay images of γH2A.X (DNA damage) and quantification of relative tail length are shown. **l**, IF of γH2A.V (DNA damage) in *DmXPO4* knockdown S2 cells. Representative images and quantification are shown (DAPI, blue; DNA damage, green). For **a**-**f**, EV, empty vector used to overexpress the corresponding protein; siCtrl, siRNA negative control with scrambled sequences; shCtrl, the vector used to express the short-hairpin construct for knocking down the expression of the corresponding protein gene. For **a**-**j**, boxes for box-plot graphs extend from the 25th to 75th percentiles and the line in the middle is plotted at the mean. Whiskers delineate all data points from minimum to maximum. **For a-f,** representative images and quantification are shown (DAPI, blue; R-loop, red; DNA damage, green), scale bar, 20 μm. For **g**, **h**, **j**, scale bar, 10 μm. For **i**, scale bar, 15 μm. n=number of cells quantified. Data are shown as mean ± SD. P values from unpaired two-sided Student's *t* test. P values were indicated in the figures. ns: not significant. Source data are provided as a Source Data file.

Supplementary Fig. 4 | Examining the relevance of splicing factors and RPA1 to nuclear accumulation ecircRNAs or ciR-loop formation. **a**, IF of R-loop, ciR-loop, and DNA damage upon *Hel25E* overexpression (OE) in *DmXPO4* knockdown S2 cells. The corresponding empty vector (EV) of overexpression was used as a negative control. Representative images and quantification are shown (R-loop, red; DNA damage, green). **b**, Validation of circRNA levels upon Sf3a1 or Srsf1 mRNA KD in 3T3 WT cells with the corresponding shRNA. Knockdown efficiency was also shown (left). **c,** Validation of circRNA levels upon SF3A1 mRNA KD in *Drosophila* S2 cells with SF3A1 dsRNA. β-gal dsRNA was used as a negative control. Knockdown efficiency was also shown (left). **d**, IF of R-loop, ciR-loop, and DNA damage upon SF3A1 KD with dsRNA in S2 cells. β-gal dsRNA was used as a negative control. Representative images and quantification are shown (R-loop, red; DNA damage, green). **e**, Validation of shRNA knockdown (KD) efficiency of RPA1 mRNA with RT-qPCR in HEK293T cells. Short-hairpin construct (shRPA1) is used for the knocking down, and shCtrl, the

negative control, is the vector of the KD construct. **f**, IF of γH2A.X (DNA damage) upon RPA1 KD in HEK293T cells. Representative images of two independent experiments are shown (DAPI, blue; DNA damage, green). **g**, IF of R-loop and ciR-loop upon RPA1 KD in HEK293T cells. Representative images and quantification are shown (DAPI, blue; R-loop, red). **h**, Representative FISH images of ecircRNA upon RPA1 in HEK293T cells Quantitation of relative FISH signals in the nucleus is shown (ecircRNA, red). For **a, d, g, h**, Boxes for box-plot graphs extend from the 25th to 75th percentiles and the line in the middle is plotted at the mean. Whiskers delineate all data points from minimum to maximum. For **a, d**, scale bar, 10 μm. For **f-h**, Scale bar, 20 μm. n = number of cells quantified. For **a-e**, **g**, **h**, data are shown as means ± SD. P values from unpaired two-sided Student's *t* test. P values were indicated in the figures, ns: not significant. Source data are provided as a Source Data file.

Supplementary Fig. 5 | Defects of *Y69A2AR.16 (ceXPO4)* worms. **a**, Scheme for the generation of *Y69A2AR.16* (*ceXPO4*) KO *C. elegans*. **b,** Validation with genomic PCR and qRT-PCR (for *ceXPO4* mRNA) of the *ceXPO4* KO in *C. elegans*. ND, not detected. Data are from three independent experiments. Western blot of the ceXPO4 protein in *C. elegans* was also performed (ACTB, Actin b as a loading control). Note: the XPO4 antibody used was not very effective in detecting ceXPO4 protein. **c**, Quantification of brood size of the *wildtype* N2 (n=22) and *ceXPO4* mutant worms (n=22). **d**, Growth rate by development stage at different time points in N2 (n=403) and *ceXPO4* mutant (n=329). **e**, Bioinformatics analyses of ecircRNAs in young adults of N2 and *ceXPO4*. Boxes for box-plot graphs extend from the 25th to 75th percentiles and the line in the middle is plotted at the median. Whiskers delineate all data points from minimum to maximum. Overlapped 245 circRNAs were analyzed. **f**, Representative IF images with enlarged regions of R-loop in the germline of N2 and *ceXPO4* mutant. Representative images are shown (DAPI, blue; R-loop, red). **g**, IF of RAD51 (examined as a dsDNA break marker) in the germline of N2 and ceXPO4 mutants. Representative images are shown (DAPI, blue; DNA damage, green). For **f**, **g**, data are representative of two independent experiments, scale bar, 30 μm. For **b**, **c**, data are shown as means ± SD. P values from unpaired two-sided Student's *t* test. For **d**, P values from the two-sided Mann-Whitney U test. For **e**, P value from two-sided Wilcox rank-sum test and were indicated in the figures. Source data are provided as a Source Data file.

Supplementary Fig. 6 | Features of liR-loop and ciR-loop in WT and XPO4 KO cells. a, Metaplots of liR-loop peaks along the gene body in different expression levels of WT and XPO4 KO cells, respectively. Genes with lower (yellow line) expression levels were compared to genes with higher expression levels (red line). **b**. Boxplot and cumulative distribution function curve show the expression level of the overlapped or immediately neighboring gene of liR-loops. **c**, Metaplot of ciR-loop peaks along the gene body in different expression levels of WT and KO cells, respectively. **d**, Boxplot and cumulative fraction curve shows the expression level of the overlapped or immediately neighboring gene of ciR-loops. P values for cumulative fraction curves in **b** (right) and **d** (right) are from Kolmogorov-Smirnov test; For **a**, **b** (left), **c** and **d** (left), P values of box plots are from unpaired two-sided Student's *t* test. P values were indicated in the figures. Boxes for box-plot graphs in **b** and **d** extend from the 25th to 75th percentiles and the line in the middle is plotted at the mean. Whiskers delineate all data points from minimum to maximum. n=number of circRNAs analyzed. Source data are provided as a Source Data file.

Supplementary Fig. 7 | Fertility and neurological defects of *XPO4*$^{+/-}$ mice. a, Mating setups with one male and two females. Offspring numbers were within 60 days of the setup. 8 *XPO4*$^{+/-}$ males were tested. 8 *XPO4*$^{+/+}$ males were used as controls. **b**, Sperm morphology in *Xpo4*$^{+/+}$ and *Xpo4*$^{+/-}$ mice. Representative abnormalities such as coiled (white arrows)/bent (red arrows) flagella, irregular shapes (blue arrow), and missing heads (orange arrows) in *Xpo4*$^{+/-}$ mice are shown. Scale bars, 50 μm. **c,** Quantitation of progressive cells and motile cells with computer-assisted sperm analysis (CASA). Sperm of *Xpo4*$^{+/-}$ mice (n=6) and *Xpo4*$^{+/+}$ mice (n=6) were examined. **d**, Representative images of Hematoxylin and Eosin (H&E) staining of *Xpo4*$^{+/+}$ and *Xpo4*$^{+/-}$ testes. Quantitation for the diameter (normalized to the *Xpo4*$^{+/+}$) of seminiferous tubule from each group (n=6) is shown. 20 seminiferous tubules from each group were analyzed. Scale bars, 100 μm. **e,** Representative images of H&E staining of *Xpo4*$^{+/+}$ and *Xpo4*$^{+/-}$ epididymis. Abnormal epididymal lumina are shown in black triangle. Scale bars, 100 μm. **f**, Percentage of spontaneous alternation (Y maze) was compared between *Xpo4*$^{+/+}$ and *Xpo4*$^{+/-}$ mice. All experiments were conducted using 4-month-old mice; n=11 (*Xpo4*$^{+/+}$) and 8 (*Xpo4*$^{+/-}$). **g**, Time (in seconds) to identify target holes for *Xpo4*$^{+/+}$ and *Xpo4*$^{+/-}$ mice in training Day 1, Day 2, Day 3 and Day 4 of the Barnes maze. All experiments were conducted using 4-month-old mice; n=11 (*Xpo4*$^{+/+}$) and 8 (*Xpo4*$^{+/-}$).

For **b**, **e**, images are representative of two independent experiments. For **c, d, f, g**, data are shown as means ± SD. P values from unpaired two-sided Student's *t* test. P values were indicated in the figures, ns: not significant. Source data are provided as a Source Data file.

Supplementary Fig. 1

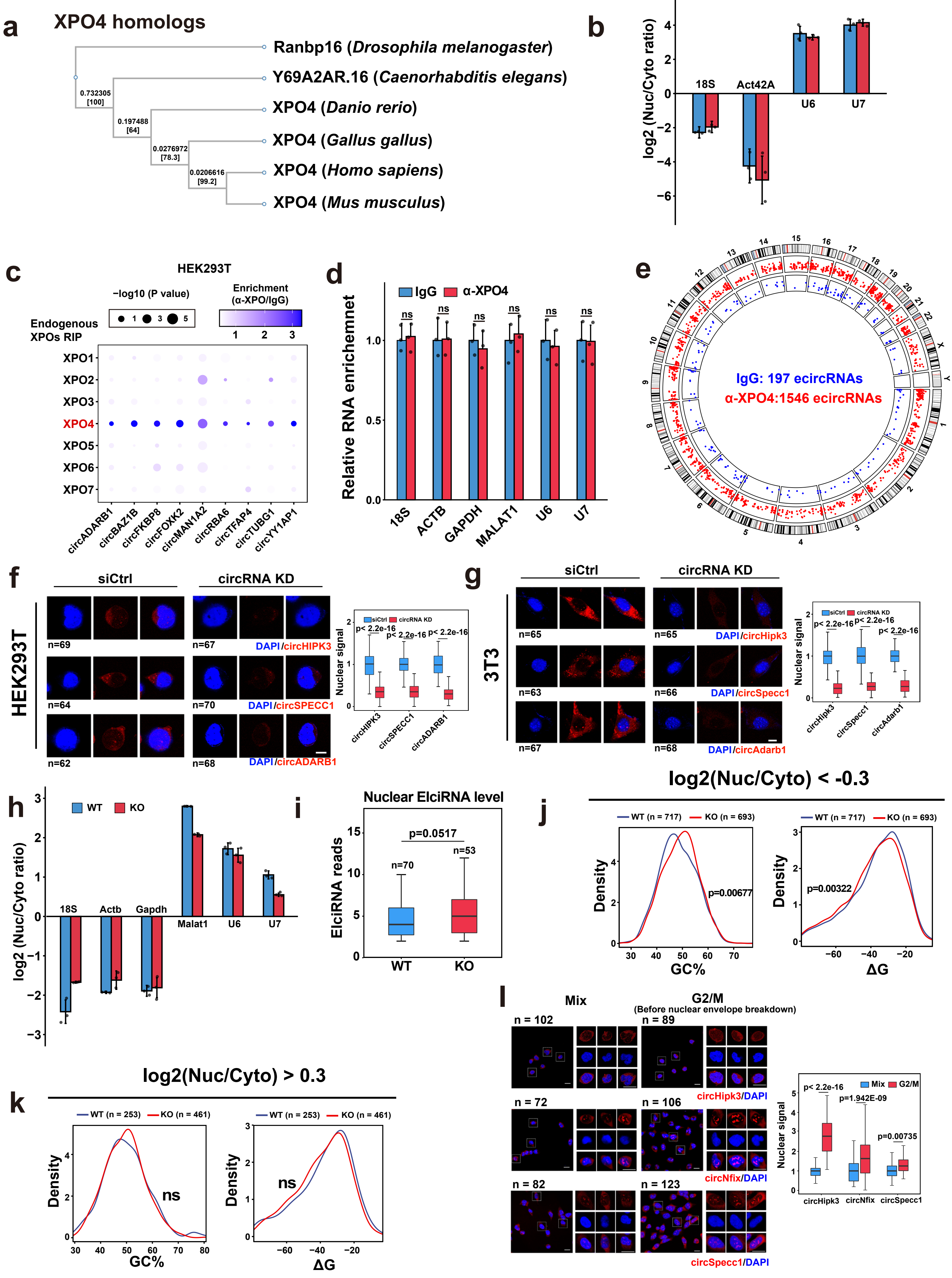

Supplementary Fig. 2

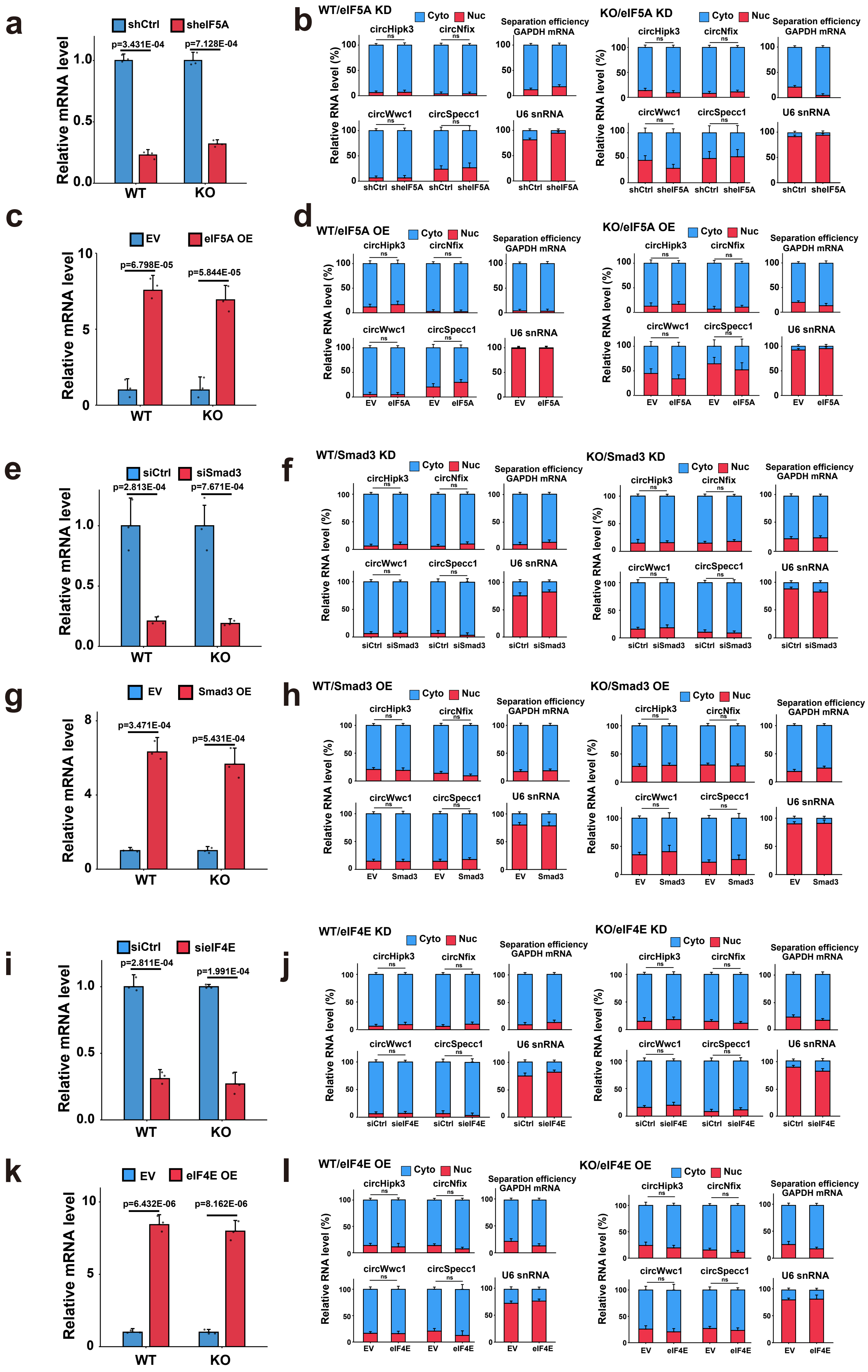

Supplementary Fig. 3

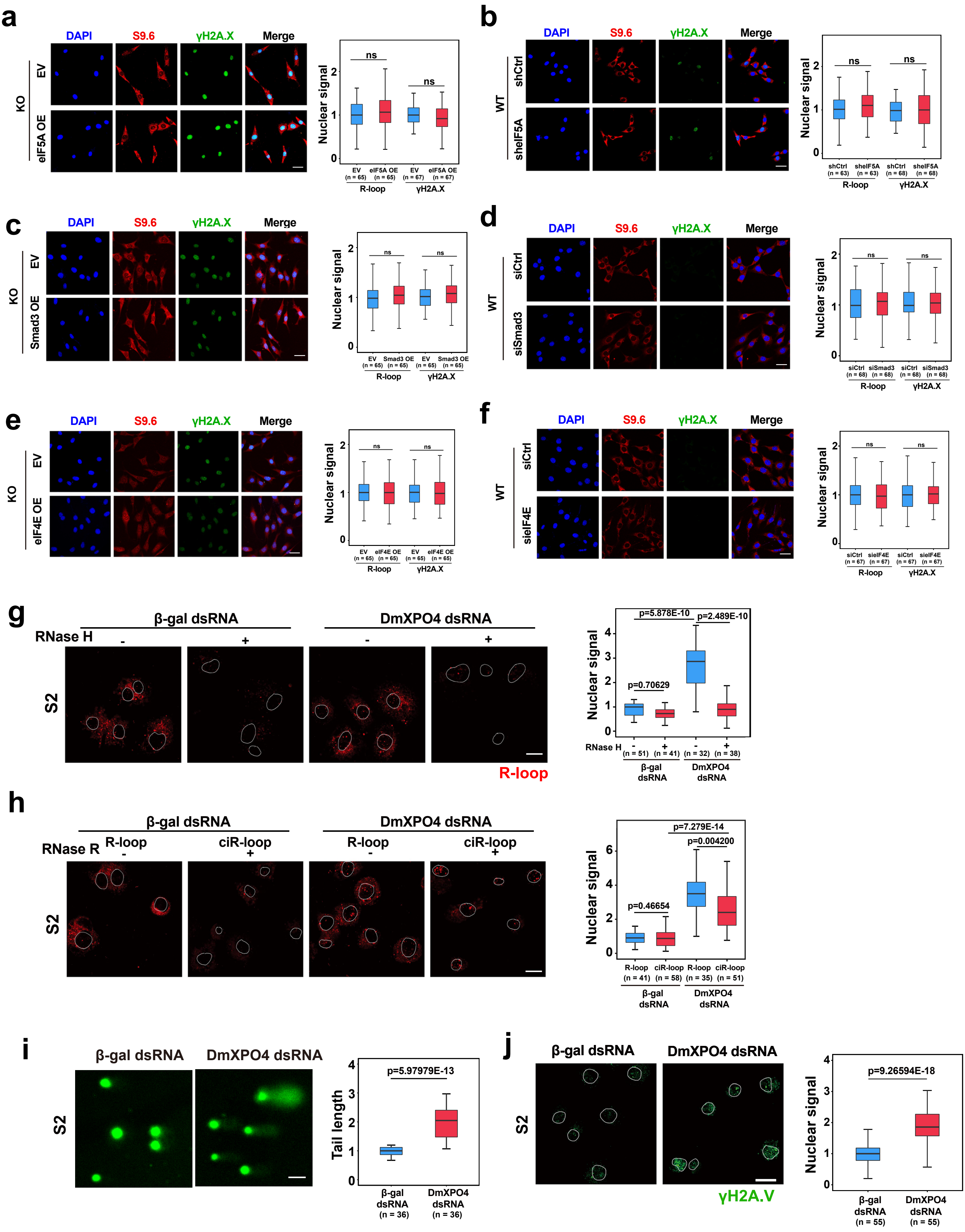

Supplementary Fig. 4

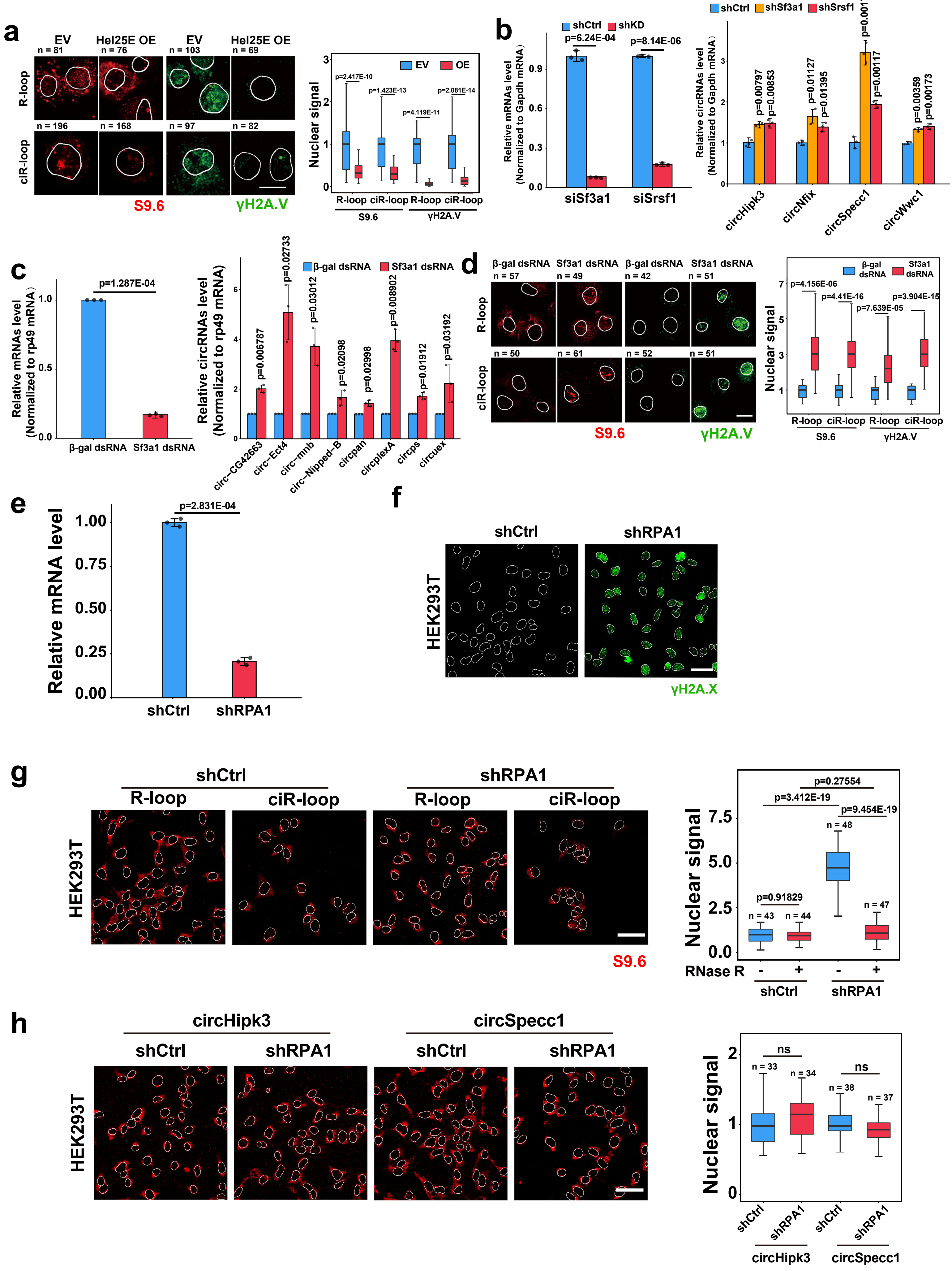

Supplementary Fig. 5

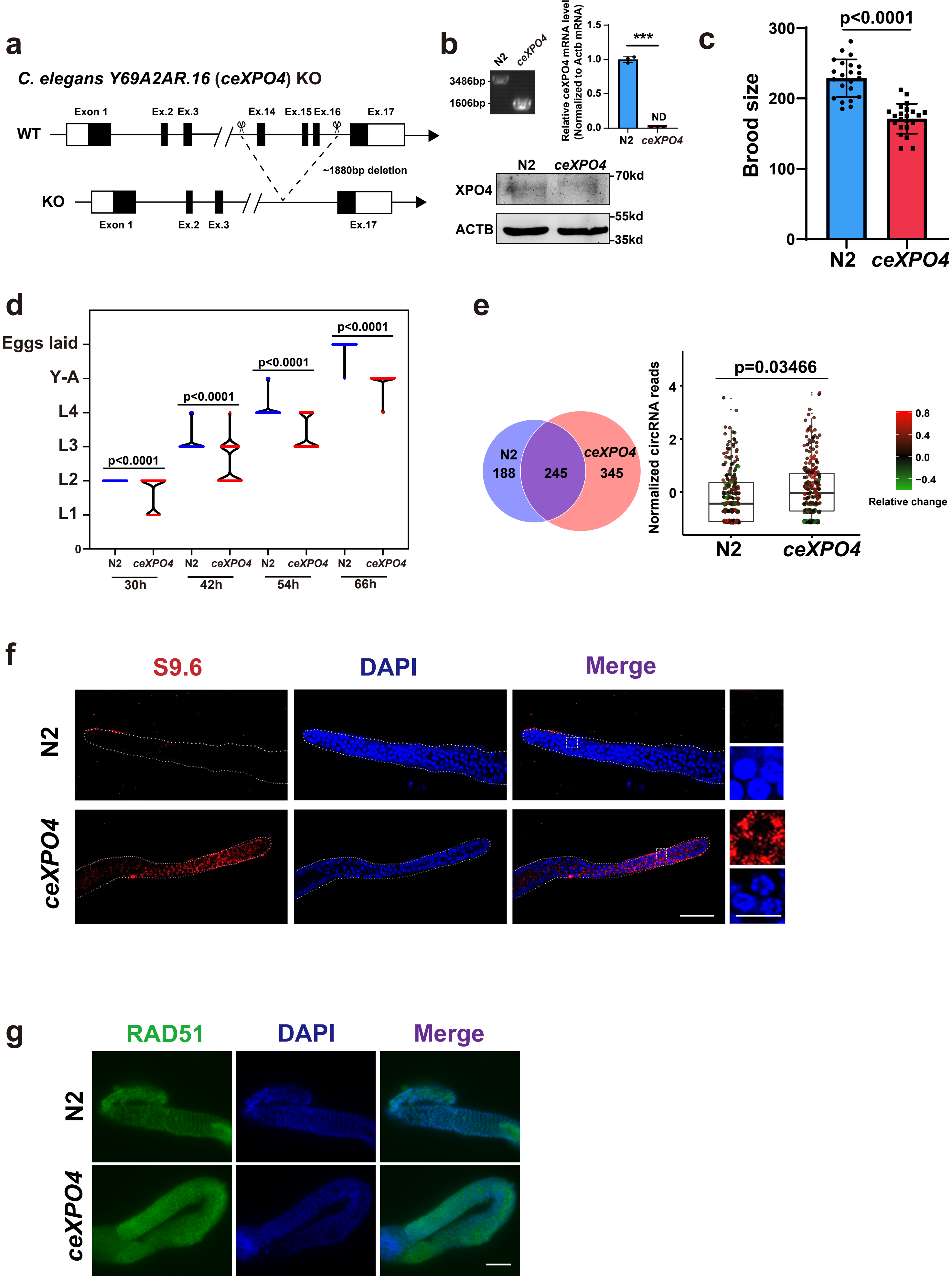

Supplementary Fig. 6

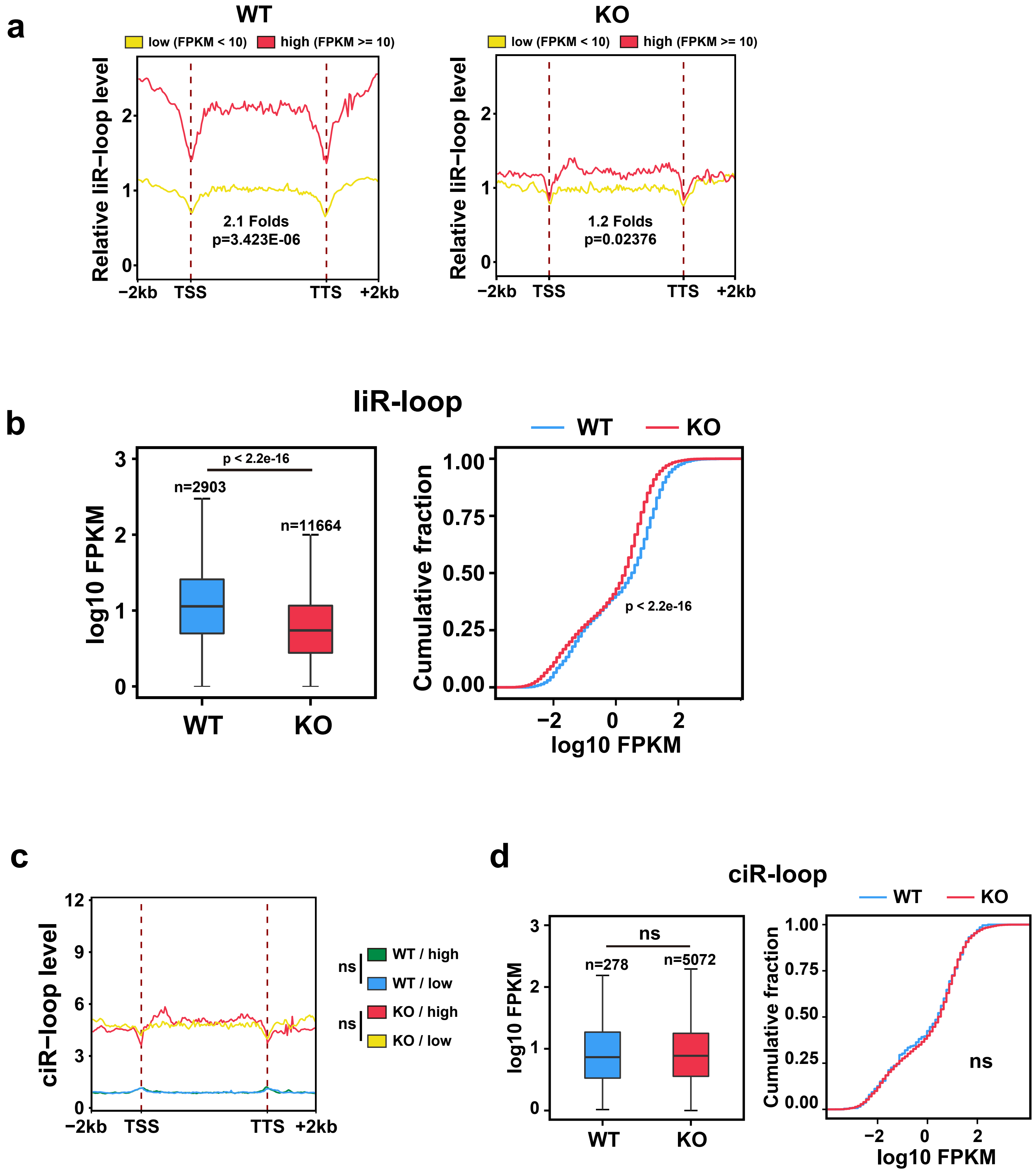

Supplementary Fig. 7

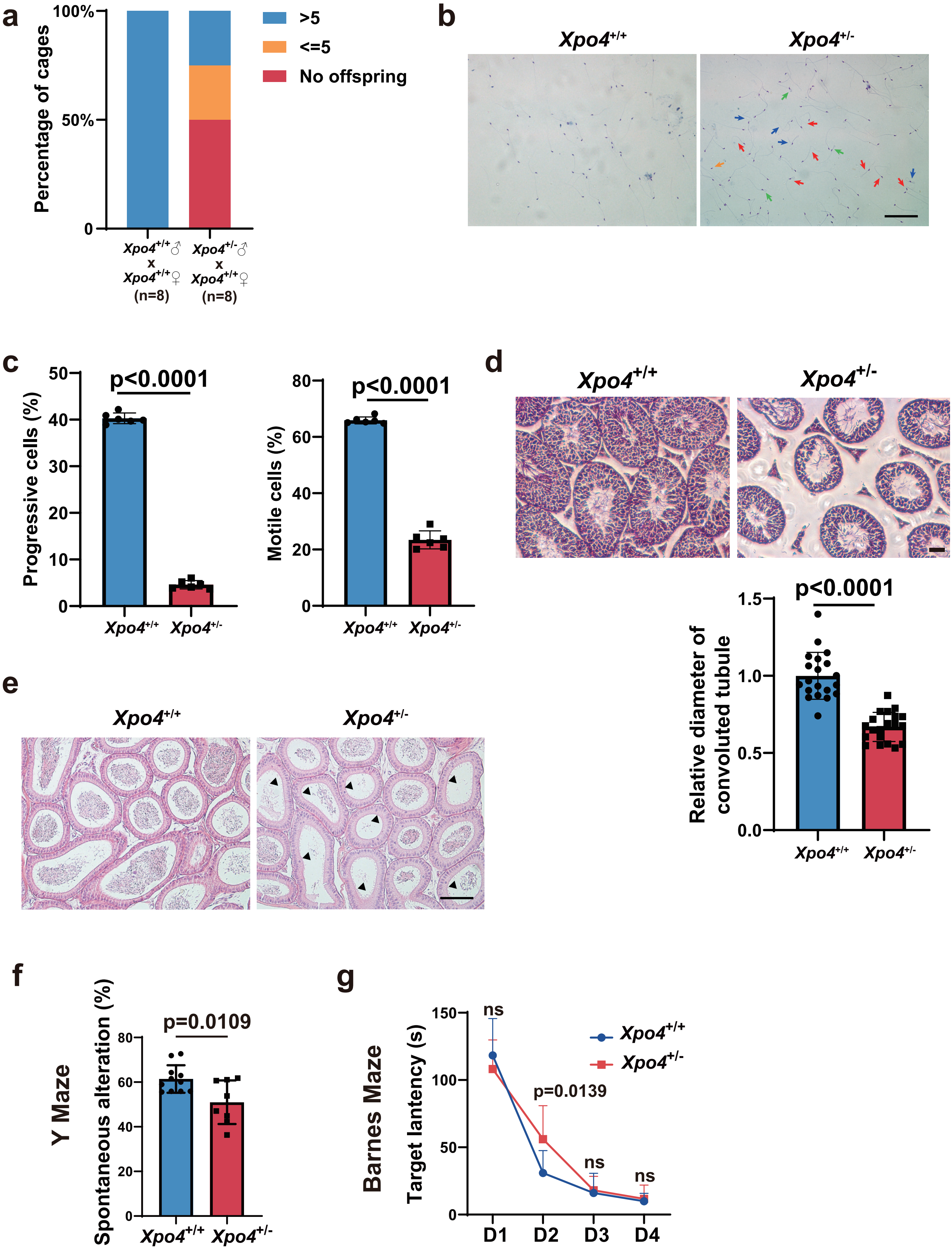

Supplement: Supplementary file 1 — Supplementary Information [file 41467_2022_33356_MOESM1_ESM.pdf]
