# Supplementary material for: Exportin 4 depletion leads to nuclear accumulation of a subset of circular RNAs
Source: Nat Commun. 2022 Oct 1;13:5769. doi: 10.1038/s41467-022-33356-z (PMC9526749; doi:10.1038/s41467-022-33356-z)
Supplement: Supplementary file 2 — Reporting Summary [file 41467_2022_33356_MOESM2_ESM.pdf]

Corresponding author(s): Ge Shan, Chuan Huang

Last updated by author(s): Aug 18, 2022

Reporting Summary

Nature Portfolio wishes to improve the reproducibility of the work that we publish. This form provides structure for consistency and transparency in reporting. For further information on Nature Portfolio policies, see our Editorial Policies and the Editorial Policy Checklist.

Statistics

For all statistical analyses, confirm that the following items are present in the figure legend, table legend, main text, or Methods section.

n/a	Confirmed	
☐	☒	The exact sample size (*n*) for each experimental group/condition, given as a discrete number and unit of measurement
☐	☒	A statement on whether measurements were taken from distinct samples or whether the same sample was measured repeatedly
☐	☒	The statistical test(s) used AND whether they are one- or two-sided<br>*Only common tests should be described solely by name; describe more complex techniques in the Methods section.*
☒	☐	A description of all covariates tested
☐	☒	A description of any assumptions or corrections, such as tests of normality and adjustment for multiple comparisons
☐	☒	A full description of the statistical parameters including central tendency (e.g. means) or other basic estimates (e.g. regression coefficient) AND variation (e.g. standard deviation) or associated estimates of uncertainty (e.g. confidence intervals)
☐	☒	For null hypothesis testing, the test statistic (e.g. *F*, *t*, *r*) with confidence intervals, effect sizes, degrees of freedom and *P* value noted<br>*Give P values as exact values whenever suitable.*
☒	☐	For Bayesian analysis, information on the choice of priors and Markov chain Monte Carlo settings
☒	☐	For hierarchical and complex designs, identification of the appropriate level for tests and full reporting of outcomes
☒	☐	Estimates of effect sizes (e.g. Cohen's *d*, Pearson's *r*), indicating how they were calculated

Our web collection on statistics for biologists contains articles on many of the points above.

Software and code

Policy information about availability of computer code

Data collection	Illumina Nova seq 6000 system at Novogene, China for RNA-seq<br>Illumina Nova seq 6000 system at Genewiz China for DRIP-seq<br>ImageQuant LAS4000 Biomolecular Imager was used to take Western blot fluorescent images.<br>Olympus IX-71 confocal microscopy was used to take cell and testis tissue fluorescent images.<br>Zeiss LSM 800 confocal microscopy was used to take brain tissue fluorescent images.
Data analysis	GraphPad Prism v 7.1<br>cutadapt v 4.1<br>Bowties2 v 2.4.1<br>Samtools v 1.10<br>HISAT2 v 2.0.5<br>featureCounts v 2.0.0<br>DEseq2 v 1.28.1<br>find_circ v 1.2<br>R software v 4.0.2<br>R studio v 1.3.1093<br>ggplot2 v.3.3.5<br>dplyr v 1.0.8<br>macs2 v 2.2.7.1

bedtools v 2.30.0
IGV v 2.8.10
FAXS v 6.0
deeptools v 3.5.1
python 3.7.1
ZEN v 2.6
Fiji (ImageJ) v1.53c

For manuscripts utilizing custom algorithms or software that are central to the research but not yet described in published literature, software must be made available to editors and reviewers. We strongly encourage code deposition in a community repository (e.g. GitHub). See the Nature Portfolio guidelines for submitting code & software for further information.

Data

Policy information about availability of data

All manuscripts must include a data availability statement. This statement should provide the following information, where applicable:

- Accession codes, unique identifiers, or web links for publicly available datasets
- A description of any restrictions on data availability
- For clinical datasets or third party data, please ensure that the statement adheres to our policy

The RNA-seq data generated in this study have been deposited in the GEO database under accession code GSE196317 [https://www.ncbi.nlm.nih.gov/geo/query/acc.cgi?acc=GSE196317]. The DRIP-seq data generated in this study have been deposited in the GEO database under accession code GSE196318 [https://www.ncbi.nlm.nih.gov/geo/query/acc.cgi?acc=GSE196318]. The processed high-throughput data are available at GEO database in same accession code. The statistics data generated in this study are provided in the Source Data file.

Human research participants

Policy information about studies involving human research participants and Sex and Gender in Research.

Reporting on sex and gender	N/A
Population characteristics	N/A
Recruitment	N/A
Ethics oversight	N/A

Note that full information on the approval of the study protocol must also be provided in the manuscript.

Field-specific reporting

Please select the one below that is the best fit for your research. If you are not sure, read the appropriate sections before making your selection.

[x] Life sciences [ ] Behavioural & social sciences [ ] Ecological, evolutionary & environmental sciences

For a reference copy of the document with all sections, see nature.com/documents/nr-reporting-summary-flat.pdf

Life sciences study design

All studies must disclose on these points even when the disclosure is negative.

Sample size	No statistical methods were used to estimate sample size. Sample size was determined based on previous studies in the field to generate reproducible results. In animal experiments, at least three animal samples were used in one group.
Data exclusions	No data was excluded from the study.
Replication	RNA-seq and DRIP-seq samples from 3T3 WT and XPO4 KO cells with two replicates. qPCR, WB, IF,IP and FISH were performed with at least 3 biological replicates. These replicate numbers were indicated in figures and figure legend. All attempts for replication were successful.
Randomization	Randomization for samples and organisms allocation is not relevant on this study because all the experiments performed with identifiable groups based on the experimental conditions(e.g. WT vs HET mouse strain, WT vs XPO4 KO 3T3 cell line). Samples within control or experimental groups were randomly assigned. In addition, the ecircRNAs were randomly chosen for imaging and validation with R script. FISH and IF quantifiaitons of signals in different areas were randomly chosen.
Blinding	The experiments were not performed blindly due to the researchers need to verify the genotype of cell line or mouse. (e.g. WT vs HET mouse strain, WT vs XPO4 KO 3T3 cell line). All comparisons were performed automatically using statistical software that is not influenced by the investigator.

Reporting for specific materials, systems and methods

We require information from authors about some types of materials, experimental systems and methods used in many studies. Here, indicate whether each material, system or method listed is relevant to your study. If you are not sure if a list item applies to your research, read the appropriate section before selecting a response.

Materials & experimental systems

n/a	Involved in the study
☐	☒ Antibodies
☐	☒ Eukaryotic cell lines
☒	☐ Palaeontology and archaeology
☐	☒ Animals and other organisms
☒	☐ Clinical data
☒	☐ Dual use research of concern

Methods

n/a	Involved in the study
☐	☒ ChIP-seq
☒	☐ Flow cytometry
☒	☐ MRI-based neuroimaging

Antibodies

Antibodies used

Antibody for immunoprecipitation
Anti-XPO1 (Santa Cruz, Cat# sc-74454; RRID:AB_1122704,3μg for IP,1:1000 for IB)
Anti-XPO2 (Santa Cruz,Cat# sc-271537; RRID:AB_1122704,3μg for IP,1:1000 for IB)
Anti-XPO3 (Santa Cruz Cat# sc-514591; RRID:AB_10649959,3μg for IP,1:1000 for IB)
Anti-XPO4 (Santa Cruz,Cat# sc-13423; RRID:AB_2215474,3μg for IP,1:1000 for IB)
Anti-XPO5 (CST,Cat #12565,3μg for IP,1:1000 for IB)
Anti-XPO6 (Proteintech, Cat# 11408-1-AP; RRID:AB_2241610,3μg for IP,1:1000 for IB)
Anti-XPO7 (Santa Cruz, Cat# sc-390025, 3μg for IP,1:1000 for IB)

Primary antibody for Western blot
Anti-XPO4 (Abcam, Cat# ab133237; RRID:AB_11155140,1:1000)
Anti-β-Actin(Transgene, Cat# HC201, RRID:AB_2860007,1:1000)
Anti-FLAG (Sigma, Cat# F1804; RRID: AB_262044,1:1000)
Anti-FLAG (used for Drosophila S2 cells, Beyotime, Cat# AF519; RRID:AB_2895204,1:1000)
Anti-α-Tubulin (Sigma, Cat# T6074;RRID:AB_477582,1:1000)
Anti-HDAC1 (Abcam, Cat# ab1767; RRID:AB_302607

Secondary antibody for Western blot
Anti-rabbit-IgG-HRP(SAB, Cat# L3012, RRID:AB_895483,1:2000)
Anti-mouse-IgG-HRP(SAB, Cat# L3032, RRID:AB_895481,1:2000)

Primary antibody for Immunofluorescence staining
Anti-NeuN (EMD millipore, Cat# MAB377; RRID:AB_2298772,1:1000)
Anti-γH2AX (used for mammalian cells, CST, Cat# 9718; RRID:AB_2118009,1:200)
Anti-γH2AX (used for Drosophila S2 cells to detect Drosophila γH2Av, Beyotime, Cat# AF5836,1:200)
S9.6 antibody (Kerafast, Cat# ENH001; RRID:AB_2687463,1:200)
Anti-XPO4 (Santa Cruz,Cat# sc-13423; RRID:AB_2215474,1:100)

Secondary antibody for Immunofluorescence staining
Anti-rabbit-IgG H&L Alexa Fluor® 488 (Abcam, Cat# ab150077, RRID:AB_2630356,1:200)
Anti-mouse-IgG H&L Alexa Fluor® 647 (Abcam, Cat# ab150075, RRID:AB_2752244,1:200)

Validation

Antibody for immunoprecipitation
Anti-XPO1: https://www.scbt.com/zh/p/crm1-antibody-c-1?requestFrom=search
Anti-XPO2: https://www.scbt.com/zh/p/cas-antibody-h-2?requestFrom=search
Anti-XPO3: https://www.scbt.com/zh/p/exportin-t-antibody-d-11?requestFrom=search
Anti-XPO4: https://www.scbt.com/zh/p/xpo4-antibody-c-1?requestFrom=search
Anti-XPO5: https://www.cellsignal.com/products/primary-antibodies/exportin-5-d7w6w-rabbit-mab/12565?site-search-type=Products&N=4294956287&Ntt=xpo5&fromPage=plp&_requestid=4010202
Anti-XPO6 :https://www.ptglab.com/products/XPO6-Antibody-11408-1-AP.htm
Anti-XPO7: https://www.scbt.com/p/exportin-7-antibody-a-11?requestFrom=search

Primary antibody for Western blot
Anti-XPO4: https://www.abcam.cn/exportin-4xpo4-antibody-epr44422-ab133237.html
Anti-β-Actin: https://www.transgen.com.cn/antibody_reference/393.html
Anti-FLAG: https://www.sigmaaldrich.cn/CN/zh/product/sigma/f1804

Anti-FLAG:https://www.beyotime.com/product/AF519.htm
Anti-α-Tubulin: https://www.sigmaaldrich.cn/CN/zh/product/sigma/t6074
Anti-HDAC1: https://www.abcam.cn/hdac1-antibody-chip-grade-ab1767.html

Secondary antibody for Western blot
Anti-rabbit-IgG-HRP: https://www.sabbiotech.com/g-170627-Goat-anti-Rabbit-IgG-Secondary-AntibodyHRP-conjugated-L3012.html
Anti-mouse-IgG-HRP: https://www.sabbiotech.com/g-170611-Goat-anti-Mouse-IgG-Secondary-AntibodyHRP-conjugated-L3032.html

Primary antibody for Immunofluorescence staining
Anti-NeuN: https://www.merckmillipore.com/HK/en/product/Anti-NeuN-Antibody-clone-A60-Alexa-Fluor488-conjugated,MM_NF-MAB377X
Anti-γH2AX: https://www.cellsignal.com/products/primary-antibodies/phospho-histone-h2a-x-ser139-20e3-rabbit-mab/9718?site-search-type=Products&N=4294956287&Ntt=anti-%CE%B3h2ax&fromPage=plp
S9.6 antibody: https://www.kerafast.com/Search?SearchTerm=s9.6

Secondary antibody for Immunofluorescence staining
Anti-rabbit-IgG H&L Alexa Fluor® 488: https://www.abcam.cn/goat-rabbit-igg-hl-alexa-fluor-488-ab150077.html
Anti-mouse-IgG H&L Alexa Fluor® 647: https://www.abcam.cn/donkey-rabbit-igg-hl-alexa-fluor-647-ab150075.html

Eukaryotic cell lines

Policy information about cell lines and Sex and Gender in Research

Cell line source(s)	HEK293T, NIH/3T3 cells and S2 cells all originate from ATCC.
Authentication	All experiments in this study used low-passage cell cultures. The morphology of each cell line was consistent with images and descriptions on the ATCC website.
Mycoplasma contamination	All cell lines were periodically confirmed to be mycoplasma-free by using the mycoplasma detection kit
Commonly misidentified lines (See ICLAC register)	No commonly misidentified line was used in this study

Animals and other research organisms

Policy information about studies involving animals; ARRIVE guidelines recommended for reporting animal research, and Sex and Gender in Research

Laboratory animals	XPO4+/- mice were generated using the CRISPR/Cas9 system with Cas9 mRNA and sgRNAs were microinjected into fertilized embryos of C57BL/6J mice. All mice were genotyped 2 weeks after birth. Deletions in XPO4 were confirmed by Sanger sequencing analysis. Specific primers for PCR were listed in the Supplementary Table 1. All XPO4+/- mice used for analyses were in parallel with age- and gender-matched wild-type littermates as a control group. Mice were housed in groups of five and were given one week to habituate before the start of the experiment upon arrival. Mice were kept in an enriched environment under the standard conditions (22±2℃ temperature, 40-60% humidity) with a 12-hour light/dark cycle (lights on from 07:00 to 19:00) at a stable temperature (23-25 °C) at the Specific-Pathogen-Free (SPF) facility. The female/male 2~5 mouth old mice were randomly assigned to experimental groups. For C. elegans culture and strains, all strains were maintained on nematode growth media (NGM) seeded OP50 at 20 ℃. N2 Bristol was obtained from the Caenorhabditis Genetic Center (CGC). All C. elegans were at the stage of young adults.
Wild animals	This study did not use wild animals.
Reporting on sex	Infertility investigations on the sperm morphology, CASA assay, HE for testis and epididymis were conducted on male mice in each group. For the neurological investigations, sex was not considered as a condition. Relevent information were listed in the Materials and Methods section "Mice" and "Sperm morphology and CASA assay".
Field-collected samples	This study does not involve field-collected samples.
Ethics oversight	All animal protocols were approved by the Animal Care and Use Committee of the University of Science and Technology of China (USTCACUC192001039).

Note that full information on the approval of the study protocol must also be provided in the manuscript.

ChIP-seq

Data deposition

- [x] Confirm that both raw and final processed data have been deposited in a public database such as GEO.
- [x] Confirm that you have deposited or provided access to graph files (e.g. BED files) for the called peaks.

Data access links
May remain private before publication.

https://www.ncbi.nlm.nih.gov/geo/query/acc.cgi?acc=GSE196317
All data was confirmed to access

Files in database submission

BioSamples:GSM5870404,GSM5870405,GSM5870406,GSM5870407,GSM5870408,GSM5870409,GSM5870410,GSM5870411,GSM5870412,GSM5870413,GSM5870414,GSM5870415,GSM5870416,GSM5870417,GSM5870418,GSM5870419
SRA:SRX14086316,SRX14086324,SRX14086325,SRX14086326,SRX14086327,SRX14086328,SRX14086329,SRX14086330,SRX14086317,SRX14086318,SRX14086319,SRX14086320,SRX14086321,SRX14086322,SRX14086323

Genome browser session
(e.g. UCSC)

https://genome.ucsc.edu/cgi-bin/hgTracks?db=mm10&lastVirtModeType=default&lastVirtModeExtraState=&virtModeType=default&virtMode=0&nonVirtPosition=&position=chr3%3A34650405%2D34652461&hgsid=1400019013_mKKSlP6KlQXF5h5jyWybqGHJgqZB

Methodology

Replicates

DRIP-seq samples from 3T3 WT and XPO4 KO cells with two replicates in each group.

Sequencing depth

DRIP-seq was performed on an Illumina Nova seq 6000 system platform with PE 150-bp reads at the Novogene, China. Each sample is sequenced with 6~10G of raw data.
KO_input_RNaseR-_rep1 has 20041078 total reads (17219294 uniquely mapped reads);
KO_input_RNaseR-_rep2 has 19985114 total reads (17143231 uniquely mapped reads);
KO_input_RNaseR+_rep1 has 20125729 total reads (17406743 uniquely mapped reads);
KO_input_RNaseR+_rep2 has total 22082483 reads (18838566 uniquely mapped reads);
KO_IP_RNaseR-_rep1 has total 33496945 reads (22067787 uniquely mapped reads);
KO_IP_RNaseR-_rep2 has total 33934817 reads (22542899 uniquely mapped reads);
KO_IP_RNaseR+_rep1 has total 32311263 reads (20000672 uniquely mapped reads);
KO_IP_RNaseR+_rep2 has total 31474282 reads (19979874 uniquely mapped reads);
WT_input_RNaseR-_rep1 has total 9621651 reads (8678729 uniquely mapped reads);
WT_input_RNaseR-_rep2 has total 11145814 reads (9914202 uniquely mapped reads);
WT_IP_RNaseR-_rep1 has total 16989008 reads (5946152 uniquely mapped reads);
WT_IP_RNaseR-_rep2 has total 18119586 reads (6044875 uniquely mapped reads);
WT_input_RNaseR+_rep1 has total 12598456 reads (11727903 uniquely mapped reads);
WT_input_RNaseR+_rep2 has total 12965756 reads (12050374 uniquely mapped reads);
WT_IP_RNaseR+_rep1 has total 25341954 reads (17655739 uniquely mapped reads);
WT_IP_RNaseR+_rep2 has total 36501205 reads (26193265 uniquely mapped reads)

Antibodies

S9.6 antibody

Peak calling parameters

-f BAMPE -q 0.001 -g mm

Data quality

The quality of the DNA reads were quality checked with FastQC . Number of peaks at FDR 5% and above 5-fold enrichment:
KO_IP_RNaseR-_rep1 has 26219;
KO_IP_RNaseR-_rep2 has 32031;
KO_IP_RNaseR+_rep1 has 6372;
KO_IP_RNaseR+_rep2 has 5996;
WT_IP_RNaseR-_rep1 has 3885;
WT_IP_RNaseR-_rep2 has 2171;
WT_IP_RNaseR+_rep1 has 271;
WT_IP_RNaseR+_rep2 has 352

Software

DRIP seq profiles were analyzed using Bowtie2(version 2.4.1) for alignment and MACS(version 2.2.7.1)for peakcalling. The bamCoverage pipeline in deeptools (v 3.5.1) were used to generate R-loop reads coverage files (.bw). The .bw files from two replicates in each group was merged by bigwigCompare pipeline (-operation mean) for IGV visualization. The common peaks from the replicates were merged using bedtools (v2.30.0). DRIP-seq data described in this study are deposited in the GEO with the accession number GSE196317.
